# ﻿Comparative and phylogenetic analyses using mitogenomes revealed gene re-arrangement of *Boletaceae* (*Boletales*)

**DOI:** 10.3897/imafungus.16.154192

**Published:** 2025-08-15

**Authors:** Xianyi Wang, Jiawei Tao, Zhongyao Guo, Guoyu Wang, Guangyin Xu, Yaping Wang, Yaohang Long, Hongmei Liu

**Affiliations:** 1 Engineering Research Center of Medical Biotechnology, School of Biology and Engineering, Guizhou Medical University, Guiyang, 561113, China Guizhou Medical University Guiyang China; 2 School of Public Health, The Key Laboratory of Environmental Pollution Monitoring and Disease Control, Ministry of Education, Guizhou Medical University, Guiyang, 561113, China Engineering Research Center of Health Medicine Biotechnology of Institution of Higher Education of Guizhou Province Guiyang China; 3 Engineering Research Center of Health Medicine Biotechnology of Institution of Higher Education of Guizhou Province, Guiyang, China Guizhou Medical University Guiyang China; 4 Key Laboratory of Biology and Medical Engineering, Immune Cells and Antibody Engineering Research Center of Guizhou Province, School of Biology and Engineering, Guizhou Medical University, Guiyang, 561113, China Engineering Research Center of Health Medicine Biotechnology of Institution of Higher Education of Guizhou Province Guiyang China; 5 The High Efficacy Application of Natural Medicinal Resources Engineering Center of Guizhou Province (The Key Laboratory of Optimal Utilization of Natural Medicine Resources), School of Pharmaceutical Sciences, Guizhou Medical University, Guiyang, 561113, China Guizhou Medical University Guiyang China; 6 School of Basic Medicine Science, Guizhou Medical University, Guiyang, 561113, China Engineering Research Center of Health Medicine Biotechnology of Institution of Higher Education of Guizhou Province Guiyang China

**Keywords:** *Boletaceae*, evolutionary rates, conserved gene clusters, injury discolouration

## Abstract

*Boletaceae*, the largest family in the *Boletales* order, is an ecologically and economically important group and the phylogenetic studies of this group need to be further developed. The mitogenome is an effective molecular marker for analysing phylogenetic relationships; however, *Boletaceae* mitochondrial genome (mitogenome) has been studied to a lesser extent. Thus, a comparative analysis of the mitogenomic features of seven *Boletaceae* species, representing seven distinct genera, was conducted. Phylogenetic relationships amongst these species within *Boletales* were reconstructed, based on the mitogenomic data. Highly consistent phylogenetic results within 34 *Boletales* species and two outgroups from *Polyporales*, based on mitogenomic datasets, were obtained using Maximum Likelihood and Bayesian Inference methods. Results of phylogenetic analyses revealed that *Boletus*, *Retiboletus* and *Neoboletus* were polyphyletic. Interestingly, species of *Neoboletus* with different bruising discolouration patterns were found in separate clades, suggesting this trait may reflect underlying genetic divergence. Furthermore, comparative and phylogenetic analyses revealed gene re-arrangements in mitogenomes of *Boletaceae*. This study is the first to report on complete mitogenomes of four genera (*Amoenoboletus*, *Hourangia*, *Leccinum* and *Strobilomyces*) and will help better understand the phylogenetic relationships of *Boletales*. Furthermore, addition of more new taxa is necessary to reconstruct a high-resolution tree.

## ﻿Introduction

The *Boletaceae* family is rich in species diversity and lifestyle and belongs to the *Boletales* order (*Fungi*, *Basidiomycota*, *Agaricomycetes*). It commonly consists of fleshy-pored boletes and many species immediately stain blue when the surface of the tubes or the context is injured. Some species of *Boletaceae* have antioxidant, anti-inflammatory and anticancer activities and serve as potential functional foods or medicines (Taofiq et al. 2015; Kim et al. 2017; Liu et al. 2023; Tian et al. 2023). Most species of this family form ectomycorrhizal relationships with vascular plants and play an important role in ecosystems (Zak et al. 2019; Bai et al. 2021). Some species can also cause a serious health hazard because of their toxic substances or exposure to metallic elements (Sha et al. 2011; Keller et al. 2018; Barea-Sepúlveda et al. 2022). During the past years, researchers worldwide have focused on identifying *Boletaceae* species, based on morphological features. However, it remains difficult to distinguish these boletes (Zang 2006; Li et al. 2021; Tao et al. 2024). The species of *Boletaceae* are widely distributed with diverse and complex structures. Moreover, the reports of new species have made identification more difficult. Continuing advances in molecular technology can help determine the phylogenetic relationships of the members of *Boletaceae* to some extent and identify them.

Several molecular markers, including internal transcribed spacer (*ITS*), 28S rDNA (*28S*), translation elongation factor 1α (*tef1α*), mitochondrial ATPase subunit 6 (*ATP6*), RNA polymerase II largest subunit (*rpb1*) and RNA polymerase II second largest subunit (*rpb2*), have been used to reconstruct the phylogenetic relationships within *Boletaceae* (Wu et al. 2014; Biketova et al. 2022; Wang et al. 2022; Wang et al. 2024). However, these sequences contain limited genetic information for interpreting relationships amongst the vast genetic diversity within *Boletaceae*. Consequently, more effective and accurate molecular markers are needed to assess the relationships between different *Boletaceae* species. The mitochondrial genome (mitogenome) is a widely studied genomic marker, exhibiting a higher evolutionary rate and more conserved transcription products than nuclear genes, making it valuable for fungal phylogenetic studies (Li et al. 2019a; Wang et al. 2020; Zhang et al. 2022; Tao et al. 2024; Wang et al. 2025). Compared to individual genes, complete mitogenomes provide both richer genetic information and diverse genomic-level characteristics (Zhang et al. 2023). Furthermore, mitochondrial gene arrangement and tRNA structure offer useful insights for determining interspecies phylogenetic relationships (Han et al. 2022; Shi et al. 2023).

The rapid development of the next-generation sequencing (NGS) technology makes it possible to obtain mitogenomes of species of *Boletaceae* easily (Li et al. 2021; Cho et al. 2022; Shi et al. 2022; Huang et al. 2024; Mu et al. 2024; Zeng et al. 2024; Zheng et al. 2024). As currently circumscribed, there are 142 genera and 1400 species of *Boletaceae* worldwide (Tremble et al. 2024). However, the number of mitogenomes of *Boletaceae* that have been published is very small. To date, only 19 mitogenomes of *Boletaceae* have been stored at the National Center for Biotechnology Information (NCBI; https://www.ncbi.nlm.nih.gov/).

In this study, seven species of *Boletaceae* collected from Guizhou Province, China, were sequenced. They are *Amoenoboletusgranulopunctatus* (Hongo) G. Wu, E. Horak and Zhu L. Yang (Wu et al. 2022), *Hourangianigropunctata* (W.F. Chiu) Xue T. Zhu and Zhu L. Yang (Zhu et al. 2015), *Retiboletusfuscus* (Hongo) N.K. Zeng and Zhu L. Yang (Zeng et al. 2016), *Leccinumparascabrum* X. Meng, Yan C. Li and Zhu L. Yang (Meng et al. 2021), *Boletusbainiugan* Dentinger (Dentinger 2013), *Strobilomycesdensisquamosus* L.H. Han and Zhu L. Yang (Han et al. 2020) and *Tylopilusneofelleus* Hongo (Hongo 1967). This study aimed to: (1) describe the mitogenomic compositions of the abovementioned seven species of *Boletaceae*; (2) compare the 26 available mitogenomes of *Boletaceae*; and (3) evaluate the phylogenetic relationships of *Boletales* with the complete mitochondrial genome of *Boletales*. This study is the first to report on mitogenomes of the genera *Amoenoboletus*, *Hourangia*, *Leccinum* and *Strobilomyces*. Mitogenomes allowed the characterisation of genomes, thereby promoting the understanding of the phylogeny, evolution and taxonomy of *Boletaceae*.

## ﻿Materials and methods

### ﻿Sample collection, DNA isolation and sequencing

The fruiting bodies of the seven species of *Boletaceae* were collected from Guizhou, China (Suppl. material 1: table S1). After examination and description, the dried specimens were deposited at Guizhou Medical University. The seven species were taxonomically identified, based on morphological characteristics and molecular markers. The total DNA was extracted using the Fungi Genomic DNA Extraction Kit (Solarbio, Beijing, China) according to the manufacturer’s instructions. The DNA samples were stored at −20°C until sequencing. The raw data of these species were obtained via NGS (Illumina HiSeq 4000 and 6 Gb raw data; Berry Genomic, Beijing, China).

### ﻿Assembly, genome annotation and sequence analysis

Mitogenomes of seven species were assembled using Geneious software. The assembled mitochondrial gene sequences were compared to the homologous sequences of *Aureoboletusraphanaceus* (Mu et al. 2024) and *Pulveroboletusravenelii* (Cho et al. 2022) retrieved from GenBank and identified through BLAST searches in the NCBI to confirm sequence accuracy. Mitogenomes were annotated with MITOS on the Galaxy platform (Galaxy Community 2022) and genetic code table 4 (Mold Protozoan mitochondrial) and MFannot (Lang et al. 2023). The locations of 15 protein-coding genes (PCGs) were confirmed using ORF Finder in Geneious (https://www.geneious.com). In particular, *atp8* was manually characterised for all newly-sequenced mitogenomes. Finally, all genes with references were compared to verify their validities. A circular mitogenomic map was created using OGDraw version 1.3.1 (Greiner and Lehwark 2019). Relative synonymous codon usage of 15 PCGs was analysed using MEGA 7 (Kumar et al. 2016). Strand asymmetry was calculated using the following formulas: AT skew = (A − T) / (A + T) and GC skew = (G − C) / (G + C) (Perna and Kocher 1995). To elucidate mitochondrial genomic evolution within *Boletaceae*, non-synonymous substitution rates (Ka) and synonymous substitution rates (Ks) of 15 core PCGs (*atp6*, *atp8*, *atp9*, *cox1*, *cox2*, *cox3*, *cob*, *nad1*, *nad2*, *nad3*, *nad4*, *nad4l*, *nad5*, *nad6* and *rps3*) in 26 mitogenomes of *Boletaceae* were calculated using DnaSP version 6.12.03 (Rozas et al. 2017). Gene synteny analysis of 26 mitogenomes of *Boletaceae* was performed using Mauve version 2.4.0 in Geneious primer (Darling et al. 2004).

### ﻿Phylogenetic analysis

The phylogenetic analyses included complete mitogenome sequences of 34 *Boletales* species. Complete mitogenomes of two *Polyporales* species (*Trametescoccinea* and *Ganodermalingzhi*, Suppl. material 1: table S2) were used as outgroups (Li et al. 2019b; Chen et al. 2021). For distinguishing, MW308606 was labelled as *Boletus* sp1 and MW308608 as *Boletus* sp2 (Li et al. 2021). The sequences of 15 PCGs (*atp6*, *atp8*, *atp9*, *cox1*, *cox2*, *cox3*, *cob*, *nad1*, *nad2*, *nad3*, *nad4*, *nad4l*, *nad5*, *nad6* and *rps3*) and two ribosomal RNAs (rRNAs; *rrnl* and *rrns*) were used to analyse the phylogenetic relationships within *Boletales*. Each PCG and rRNA sequence was aligned using the MAFFT algorithm in PhyloSuite and the MAFFT version 7.0 online service with the G-INS-i strategy, respectively (Katoh et al. 2019; Zhang et al. 2020). Poorly-aligned sites were removed using Gblocks 0.91b (Dereeper et al. 2010) under default settings. Subsequently, the resulting 17 alignments were assessed and manually corrected using MEGA 7 (Kumar et al. 2016). Five datasets were obtained, namely, PCG (all codon positions of 15 PCGs), PCG12 (first and second codon positions of 15 PCGs), PCGRNA (PCG and 2 rRNAs), PCG12RNA (PCG12 and 2 rRNAs) and AA (amino acid sequences of 15 PCGs). To test whether the five datasets were suitable for high-level phylogenetic inferences, heterogeneity in nucleotide divergence was evaluated via pairwise comparisons in a multiple sequence alignment using AliGROOVE version 1.05 (Kück et al. 2014).

Phylogenetic trees were reconstructed using Maximum Likelihood (ML) and Bayesian Inference (BI) analyses. ML analysis and the best-fit model predictions were conducted in IQ-TREE version 1.6.12 with 10,000 bootstrap replications performed using the ultrafast bootstrap method to estimate node support (Nguyen et al. 2015). The cpREV + F + R4 Model was applied to the AA dataset in the tree-building process and the GTR + F + R4 model was applied to the others. Moreover, before reconstructing the phylogenetic trees of BI, the best-fit model for each dataset was predicted using ModelFinder (Kalyaanamoorthy et al. 2017). Model cpREV + F + I + G4 was applied to the AA dataset in the tree-building process and model GTR + F + I + G4 was applied to the remaining four datasets. BI trees were constructed using MrBayes 3.2.7 (Ronquist et al. 2012), which simulate four independent runs for 1 million generations with sampling every 1000 generations. After the average standard deviation of split frequencies decreased to < 0.01, the initial 25% of the samples were discarded as burn-in and the remaining trees were used to generate a consensus tree and calculate posterior probabilities. Finally, the phylogenetic trees were visualised using FigTree version 1.4.4 (http://tree.bio.ed.ac.uk/software/figtree/) and enhanced using Adobe Illustrator CC version 22.1.

### ﻿Abbreviations

**ML** Maximum Likelihood method

**BI** Bayesian Inference method

***ITS*** internal transcribed spacer

***28S****28S* rDNA

***tef1α*** translation elongation factor 1α

***ATP6*** mitochondrial ATPase subunit 6

***rpb1*** RNA polymerase II subunit 1

**tRNAs** transfer RNAs

**NGS** the next-generation sequencing technology

**NCBI** the National Center for Biotechnology Information

***rrnl*** ribosomal large subunit

***rrns*** ribosomal small subunit

**PCGs** protein-coding genes

**rRNA** ribosomal RNA

**Ka** non-synonymous substitution rates

**Ks** synonymous substitution rates

**AA** amino acid sequences

**Ser** serine

**Leu** leucine

**Arg** arginine

**RSCU** Relative synonymous codon usage analysis

**BPP** Bayesian posterior probability

**BS** bootstrap

## ﻿Results

### ﻿Features and PCGs of the seven *Boletaceae* mitogenomes

**Figure 1.**
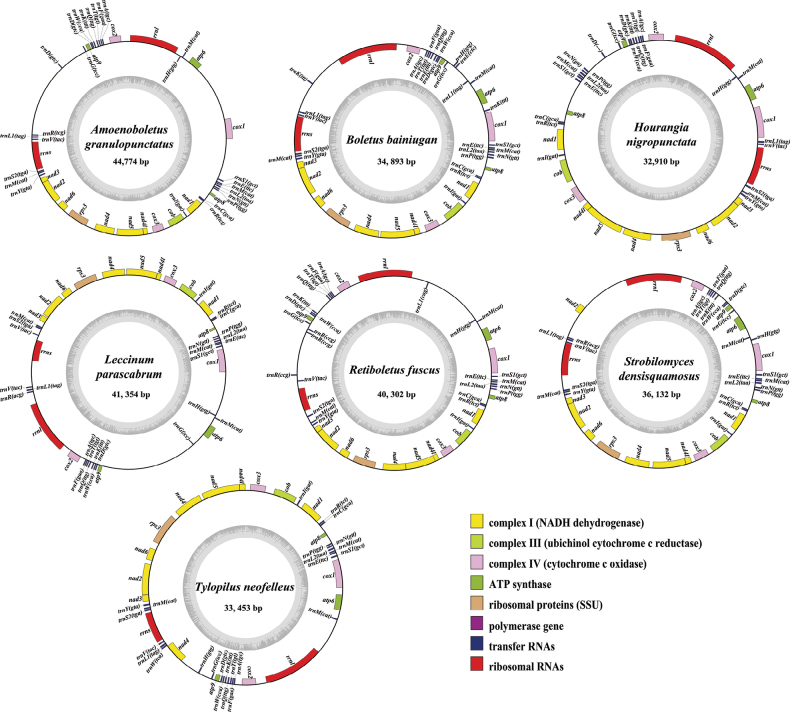
Circular maps of mitogenomes of seven newly-sequenced *Boletaceae* species. Genes are represented with different colour blocks. Strand orientation is indicated by block position: genes on the direct strand are represented by colour blocks outside the ring, while genes on the reverse strand are denoted by colour blocks inside the ring.

Complete mitogenomes of seven species of *Boletaceae*, i.e. *A.granulopunctatus* (44,774 bp in size), *H.nigropunctata* (32,910 bp in size), *R.fuscus* (40,302 bp in size), *L.parascabrum* (41,354 bp in size), *B.bainiugan* (34,893 bp in size), *S.densisquamosus* (36,132 bp in size) and *T.neofelleus* (33,453 bp in size), were composed of circular DNA molecules (Fig. 1). The sizes of the seven mitogenomes ranged from 32,910 to 44,774 bp, with an average size of 37,439 bp. The length of the mitogenome of the currently known *Boletaceae* (including those from this study and published in NCBI) ranged from 32,389 to 48,298 bp, with an average size of 38,155 bp. Amongst the seven mitogenomes sequenced, *A.granulopunctatus* contained the largest mitogenome, whereas *H.nigropunctata* contained the smallest mitogenome. The GC content of the seven mitogenomes of *Boletaceae* ranged from 22.7% to 24.1%, with an average GC content of 23.41%. *H.nigropunctata* contained the highest GC content, 24.1%, amongst the seven species investigated, which was higher than that reported for any other species of *Boletaceae* to date. The AT skews of *A.granulopunctatus*, *R.fuscus*, *B.bainiugan* and *S.densisquamosus* were positive, whereas those of the other three were negative. The GC skews in mitogenomes of most species were positive, except for *B.bainiugan*, *S.densisquamosus* and *T.neofelleus*. *L.parascabrum* contained the third-longest mitogenome amongst the seven species and consisted of three reverse repeat regions totalling 3692 bp. All seven mitogenomes contained 15 conserved core PCGs, including *atp6*, *atp8*, *atp9*, *cob*, *cox1*, *cox2*, *cox3*, *nad1*, *nad2*, *nad3*, *nad4*, *nad4l*, *nad5*, *nad6* and *rps3*. The length of the 15 PCGs ranged from 156 bp (*atp8*) to 1596 bp (*cox1*). PCG duplications were observed in *S.densisquamosus* and *T.neofelleus*. The characteristics of mitogenomes of the seven species are listed in Table 1.

**Table 1.** Characteristics of mitogenomes of seven *Boletaceae* species.

Species name	Size of mitochondrial genome (bp)	GC rate (%)	AT skew	GC skew	standard PCGs (n)	tRNAs (n)	rRNAs (n)
*Amoenoboletusgranulopunctatus*	44,774	23.4	0.032	0.036	15	26	2
*Leccinumparascabrum*	41,354	23.6	−0.011	0.029	15	25	2
*Retiboletusfuscus*	40,302	23.2	0.036	0.032	15	27	2
*Strobilomycesdensisquamosus*	36,132	22.7	0.024	−0.033	15	25	2
*Boletusbainiugan*	34,893	23.8	0.014	−0.050	15	28	2
*Tylopilusneofelleus*	33,453	23.1	−0.033	−0.011	15	25	2
*Hourangianigropunctata*	32,910	24.1	−0.029	0.068	15	25	2

### ﻿rRNA and tRNA genes

All seven *Boletaceae* mitogenomes contained two rRNA genes: *rrns* and *rrnl*. The length of the two rRNA genes ranged from 1438 bp (*rrns* of *L.parascabrum*) to 3502 bp (*rrnl* of *B.bainiugan*). Moreover, the seven mitogenomes contained 25–28 tRNAs whose lengths ranged from 57 bp (*trnC* of *B.bainiugan*) to 86 bp (*trnL1*, *trnL2* and *trnS2* of most of the seven mitogenomes). These tRNAs were located at various scattered locations across mitogenomes. All tRNAs encoded 20 standard amino acids. All seven mitogenomes contained two tRNA genes with different anticodons that encoded serine (Ser) and leucine (Leu) and three tRNAs with the same anticodons encoding methionine. The mitogenomes of *L.parascabrum*, *R.fuscus*, *A.granulopunctatus* and *S.densisquamosus* contained two tRNAs with different anticodons encoding arginine (Arg).

### ﻿Relative synonymous codon usage (RSCU) analysis and the evolutionary rates of PCGs

**Figure 2.**
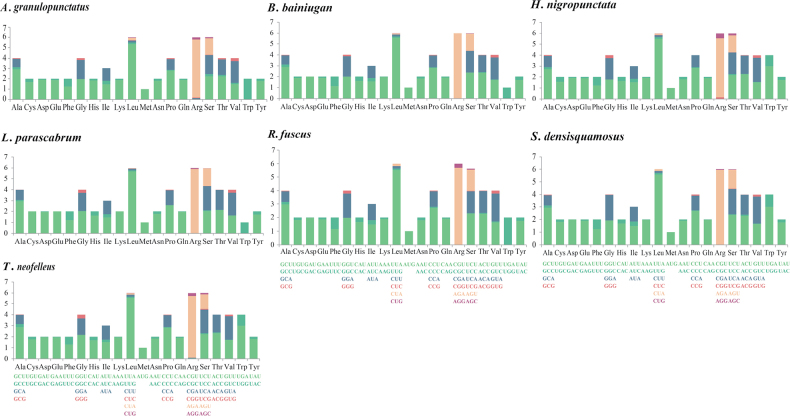
Relative synonymous codon usage analysis of 15 protein-coding genes of the seven *Boletaceae* mitogenomes. Codon families are provided on the x-axis.

Most 15 core PCGs in the seven mitogenomes used ATA, ATG, ATT and TTA as start codons, whereas *cox1*, *cox3*, *atp9* and *rps3* also used ATC, TTG and GTG as start codons. TAA was the most commonly used stop codon in mitogenomes of the seven species. In *atp8*, *nad1*, *nad2* and *nad4l*, TAG and TGA were also used. RSCU analysis indicated that the most frequently used codons in the seven mitogenomes were AGA (for Arg), UUA (for Leu), GCU (for alanine, Ala), CCU (for proline, Pro) and UCU (for Ser; Fig. 2). RSCU > 1 indicated that amino acids preferentially used the codon, whereas RSCU < 1 indicated the opposite trend. The high frequency of A and T use in codons contributed to the high AT content of the seven mitogenomes (average of 76.59%).

**Figure 3.**
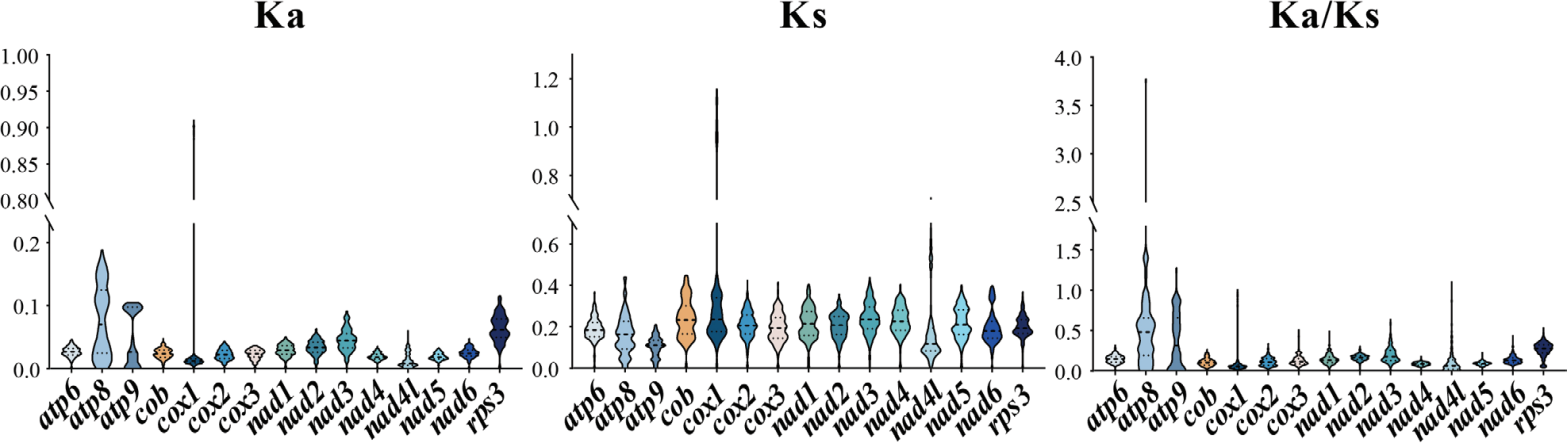
Genetic analysis of 15 core protein-coding genes amongst 26 *Boletaceae* mitogenomes. Ka, the number of non-synonymous substitutions per non-synonymous site; Ks, the number of synonymous substitutions per synonymous site.

For the 15 scrutinised core PCGs, *atp8* had the highest Ka value, whereas *nad4l* demonstrated the lowest Ka value. The Ks of *nad3* was the highest, whereas that of *atp9* was the lowest amongst the 26 species of *Boletaceae*. The Ka/Ks value of *atp8* was > 1, indicating that it was under positive selection pressure (Fig. 3). Amongst the 15 PCGs, *nad4* had the lowest Ka/Ks value and *atp8* had the highest Ka/Ks value. Some of the Ka/Ks values for *atp9* and *nad4l* were > 1, where *atp9* may be influenced by the error of Ka and *nad4l* may be influenced by a larger error in the Ks value. Overall, Ka/Ks values < 1 indicated that they were under negative selection pressure.

### ﻿Phylogenetic analyses

**Figure 4.**
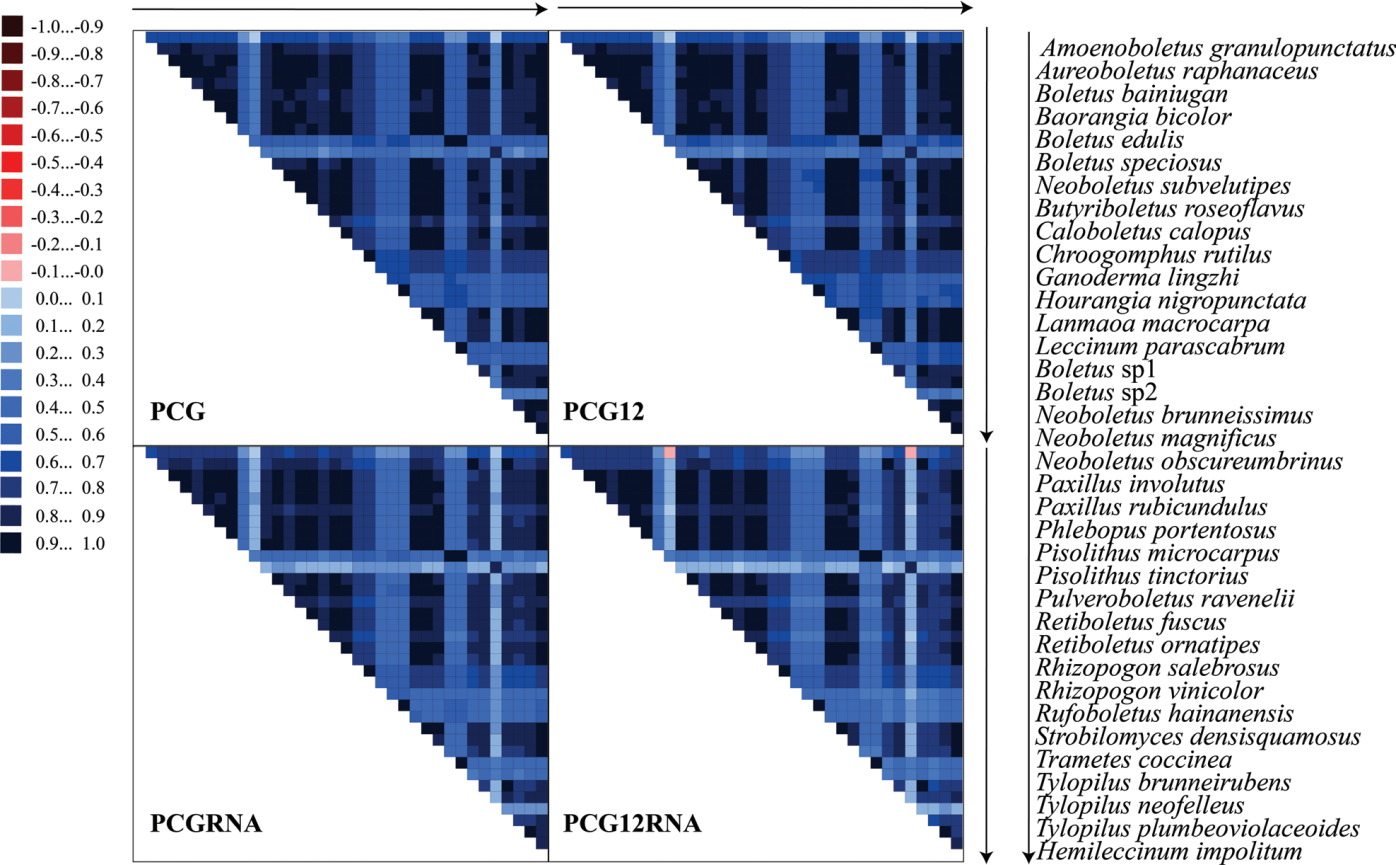
Heterogeneity of the sequence composition of mitogenomes in different datasets. The pairwise Aliscore values are indicated by coloured squares. Darker colours indicate full random similarity and lighter colours indicate the opposite. Arrows represent the order in which the species are arranged.

Datasets without ribosomal RNA sequences (PCG and PCG12) were characterised by low heterogeneity in sequence composition and higher heterogeneity was observed in the datasets with ribosomal RNA sequences (PCGRNA and PCG12RNA; Fig. 4). The dataset with the third codon removed (PCG12) showed lower heterogeneity in the two datasets without ribosomal RNA. However, the phylogenetic inferences of the PCG12RNA dataset may be biased by its high heterogeneity. The members of *Boletales* other than *Boletaceae* showed higher heterogeneity, with *Chroogomphusrutilus* having the highest heterogeneity. *A.granulopunctatus* had a slightly higher heterogeneity than other *Boletaceae* species. However, all datasets had no strong heterogeneity and were suitable for the construction of phylogenetic trees.

**Figure 5.**
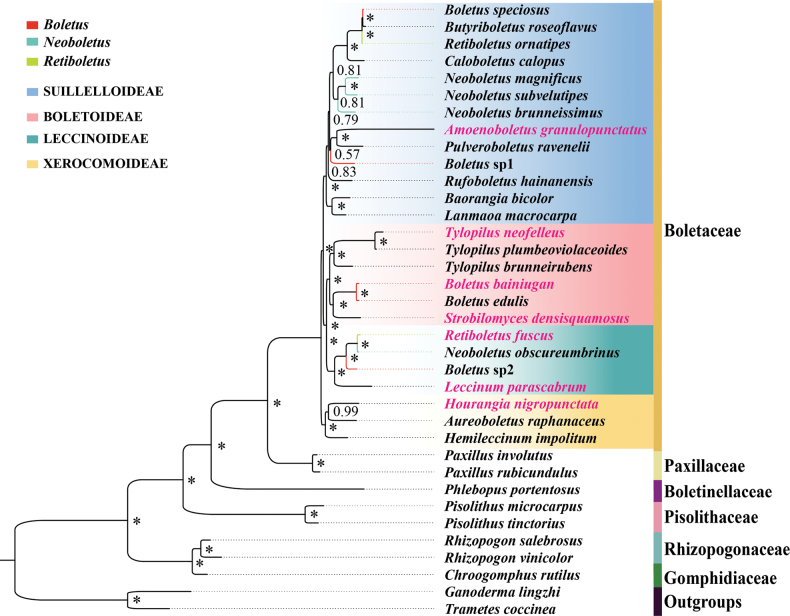
Phylogenetic relationships of 34 *Boletales*, based on AA using MrBayes. The asterisk indicates BPP = 1. The polyphyletic genus is represented by the same colour. The seven newly-sequenced mitogenomes are highlighted in pink. Four colours show the four subfamilies.

Phylogenetic analyses on five datasets (AA, PCG12, PCG12RNA, PCG and PCGRNA) of 34 species of *Boletales* and two outgroups (*T.coccinea* and *G.lingzhi*) were performed using ML and BI and ten phylogenetic trees were obtained (Fig. 5; Suppl. material 1: figs S1–S9). The main topological structures of the ten phylogenetic trees inferred by different methods were almost the same, except that there were some differences in the order of precedence in the *Boletaceae*. Based on the results of the analyses of 10 phylogenetic trees, the 34 *Boletales* species could be divided into six major clades corresponding to the families *Boletaceae*, *Boletinellaceae*, *Gomphidiaceae*, *Paxillaceae*, *Rhizopogonaceae* and *Sclerodermataceae*. A phylogenetic study, based on the mitogenome, revealed that *Boletaceae* was monophyletic. Results of the phylogenetic tree corroborated the existence of the subfamily *Suillelloideae*, thereby lending support to the recently proposed eight subfamilies of the family *Boletaceae* (Tremble et al. 2023, 2024). The monophyly of the genera *Boletus*, *Retiboletus* and *Neoboletus* was not recovered in the analyses. The monophyly of the genus *Tylopilus* was found in the topological structure produced by three datasets (AA, PCG12 and PCG12RNA). In contrast, it was not found in the topological structure produced by the other two datasets. The sister relationship between *Amoenoboletus* and *Pulveroboletus* was highly supported (Bayesian posterior probability [BPP] = 1). *Paxillaceae* species showed a closer phylogenetic relationship with *Boletaceae* species than *Boletinellaceae* species, consistent with previous studies, based on nuclear and mitochondrial genes (Wu et al. 2014; Li et al. 2021). Morphologically, *B.edulis* was similar to *B.bainiugan*. Furthermore, at the molecular level, they grouped in the phylogenetic tree with good support (BPP = 1, BS = 100).

### ﻿Gene re-arrangement

**Figure 6.**
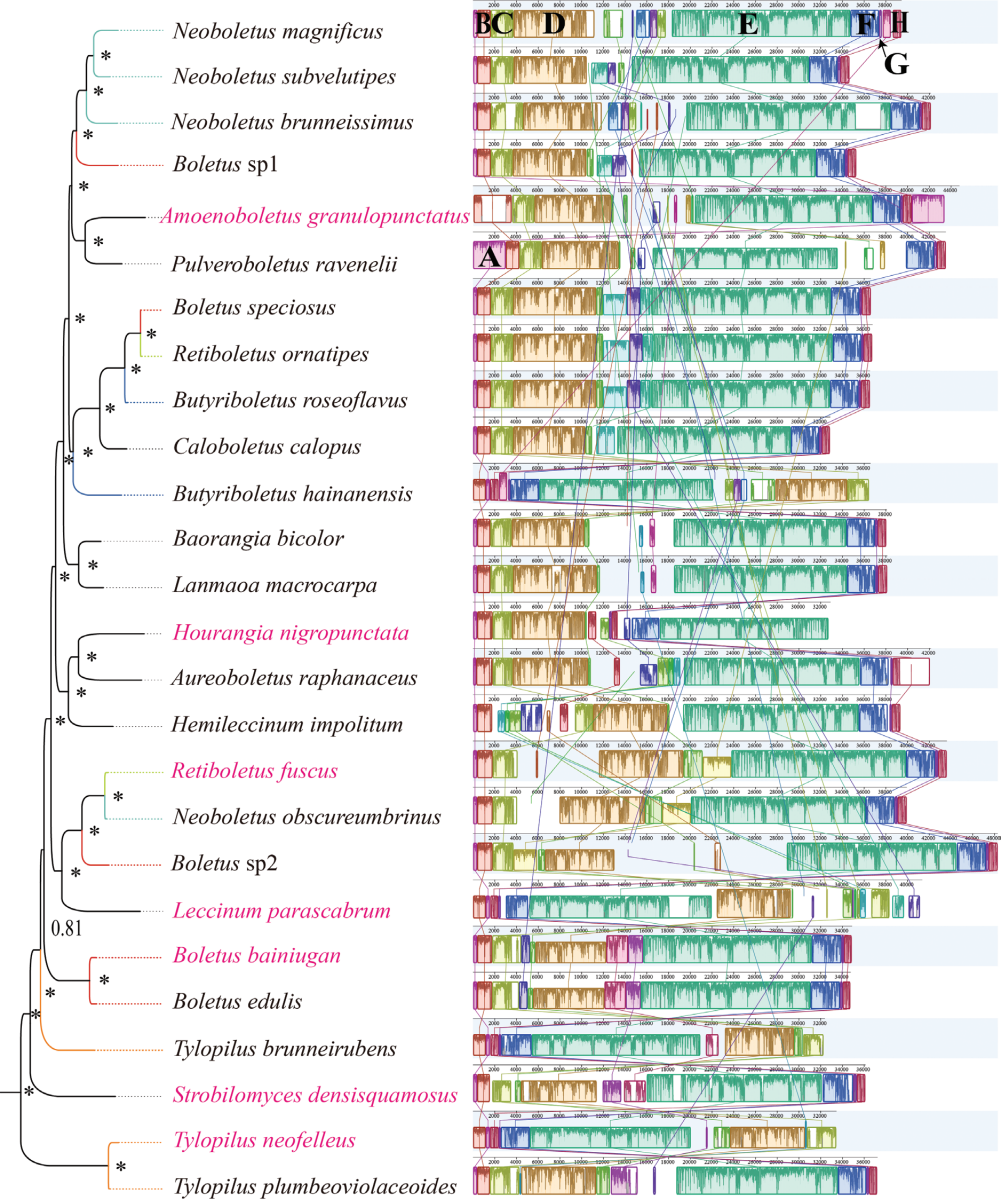
Synteny analysis of the 26 *Boletaceae* mitogenomes. Homologous regions between different *Boletaceae* species are represented by the same colour blocks and connected by the same colour lines. The asterisk indicates BPP = 1. The seven newly-sequenced mitogenomes are highlighted in pink. The polyphyletic genus is represented by the same colour.

Mitochondrial gene arrangement can be used as a reference for studying the phylogenetic status and evolution of species. Amongst all 26 mitogenomes of *Boletaceae*, six homologous regions were found, namely, A–F, using Mauve (Fig. 6). The size and location of these homologous regions varied greatly amongst the 26 species, even amongst species from the same genera. Homologous regions A–F in the genera *Boletus*, *Neoboletus* and *Retiboletus* were located at the same site, but were different in size. In the genus *Tylopilus*, the homologous regions differed in location as well as in size. There were some lineage-specific sequences in the homologous regions A, B, C, D and F. The gene arrangement of the 26 *Boletaceae* species was highly variable, indicating that *Boletaceae* had undergone large-scale gene re-arrangements during evolution.

**Figure 7.**
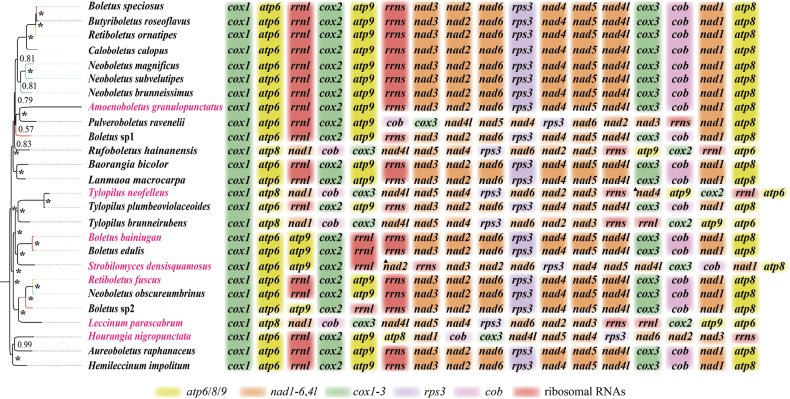
Gene order of 26 *Boletaceae* mitogenomes. The 15 core PCGs and 2 rRNA genes are included in the gene arrangement, starting from the *cox1* gene. The coloured blocks indicate different categories of genes. The asterisk indicates BPP = 1. The seven newly-sequenced mitogenomes are highlighted in pink. The polyphyletic genus is represented by the same colour. The triangles represent the duplication of this gene in the mitogenome.

The order of mitogenome genes in 26 species of *Boletaceae* was shown in Fig. 7. Fourteen of the 26 mitogenomes had the same gene order (*cox1*-*atp6*-*rrnl*-*cox2*-*atp9*-*rrns*-*nad3*-*nad2*-*nad6*-*rps3*-*nad4*-*nad5*-*nad4l*-*cox3*-*cob*-*nad1*-*atp8*). Although other species had their mitogenomes arranged in a different order, there were still three conserved gene clusters (*atp6*-*rrnl*-*cox2*-*atp9*, *rrns*-*nad3*-*nad2*-*nad6*-*rps3*-*nad4*-*nad5*-*nad4l*-*cox3*-*cob* and *nad1*-*atp8*) in their mitogenomes. Overall, the order of mitogenomic gene order of all 26 *Boletaceae* species were characterised by the presence of these three conserved gene clusters, which were arranged in a certain order. In the polyphyletic group *Boletus* only the order of the genes in *Boletus* sp2 was different. In polyphyletic groups, *Neoboletus* and *Retiboletus*, their genes were arranged in the same order. However, in the genus *Tylopilus*, all three species had their mitogenomes arranged differently. *Nad2* and *nad4* were duplicated in *S.densisquamosus* and *T.neofelleus*, respectively.

### ﻿Data availability

The seven newly-sequenced mitogenomes were deposited at GenBank under the following accession numbers: PP048750–PP048756. *T.neofelleus* (PP048750); *H.nigropunctata* (PP048751); *L.parascabrum* (PP048752); *A.granulopunctatus* (PP048753); *B.bainiugan* (PP048754); *S.densisquamosus* (PP048755); *R.fuscus* (PP048756).

## ﻿Discussion

### ﻿Variation in mitogenome size

Accumulating inverse repetitive sequences and dynamic changes in plasmid-derived regions, introns and intergenic regions were found to be the main factors contributing to size variations in fungal mitogenomes (Sandor et al. 2018). The newly-formed two longest mitogenomes in our study, *A.granulopunctatus* and *L.parascabrum*, exhibited significantly longer intergenic regions than the other mitogenomes. In addition, three pairs of inverted repeat sequences of up to 1846 bp were identified in *L.parascabrum*, which contributed to its considerable length. The length of the mitogenome of *Boletus* sp2 was the longest of those known to date in the *Boletaceae* (Li et al. 2021). A pair of 8070 bp identically orientated repeat sequences existed in its mitogenome. This indicates that the length of the repetitive sequence plays a significant role in determining the length of the mitogenome. An intron was identified in mitogenomes of the *Boletaceae*, specifically in the *cox1* gene of *P.ravenelii* (Cho et al. 2022). This results in this species having the longest *cox1* gene and the third-longest mitogenome within the *Boletaceae* up to now. In the mitogenome of *A.raphanaceus*, a polymerase gene (*dpo*) encoding a family B DNA polymerase was found, indicating the integration of mitochondrial plasmids into the mitochondrial genome (Yang et al. 2016). Furthermore, the mitogenome of *A.raphanaceus* was ranked fourth in length due to the influence of *dpo.* In combination, introns, *dpo*, repetitive sequence and intergenic regions represent the four most significant factors influencing mitochondrial genome expansion in species belonging to the *Boletaceae*.

### ﻿Phylogenetic consistency and gene order

Consistent topological patterns emerged across five mitogenomic datasets (AA, PCG12RNA, PCG12, PCG, PCGRNA) in *Boletaceae* phylogenies. Third-codon positions appeared to affect resolution, as evidenced by conflicting support for *Tylopilus* monophyly: datasets including third codons (PCG/PCGRNA; Suppl. material 1: figs S2–S5) failed to support it, whereas exclusion of these positions (AA/PCG12/PCG12RNA; Fig. 5; Suppl. material 1: figs S1, S6–S9) yielded monophyletic groupings. This inconsistency may relate to potential saturation effects in third-codon positions (Breinholt and Kawahara 2013). Gene arrangements have largely remained conserved, as evidenced by the presence of three conserved gene clusters. Although *B.bainiugan* and *B.edulis* have slight morphological differences in pileal margin and basidiospore size (Cui et al. 2016), their gene orders are almost identical. Inconsistency in phylogenetic results of *Tylopilus* may also be related to the order of mitochondrial genes. The arrangement of their mitochondrial genes varies.

### ﻿Taxonomic implications of polyphyletic genera

Multiple genera exhibited non-monophyletic distributions: *Boletus*, *Retiboletus* and *Neoboletus* showed polyphyletic patterns across datasets, consistent with earlier morphological and molecular reports (Li et al. 2021). Bruising-induced discolouration may display phylogenetic correlation, with intensely blue-bruising species grouping with similarly reactive *N.subvelutipes*, while slower-discolouring taxa grouped with mildly reactive *R.fuscus* and *L.parascabrum* (Yang et al. 2023). This pattern suggests bruising reactivity may align with evolutionary divergences in *Neoboletus*. Conversely, *Tylopilus* monophyly received inconsistent support, contradicted by third-codon-inclusive mitogenomic data and prior nuclear and morphological analyses, though a sister relationship between *T.neofelleus* and *T.plumbeoviolaceoides* was observed despite historical taxonomic complexities (Gelardi et al. 2015; Li and Yang 2021). Given *Boletaceae*’s ecological and economic importance as ectomycorrhizal symbionts and edible fungi (Wang et al. 2014), future studies combining expanded global sampling with integrated mitogenomic, nuclear and morphological approaches may help clarify these taxonomic uncertainties.

## ﻿Conclusions

This study identified mitogenomes of seven species of *Boletaceae* and comparatively described their characterisation. Two factors, repetitive sequence and intergenic regions, were found to substantially influence the mitogenome size in the seven species. In the mitogenome of the *Boletaceae*, one PCG experienced positive selection pressure and 14 PCGs experienced negative selection pressure; *atp8* had the highest Ka/Ks value. Six homologous regions and the gene order of mitogenomes of *Boletaceae* were also detected. Three conserved gene clusters were found in mitogenomes of species in the *Boletaceae*. The known mitogenomes were all of these three gene clusters arranged in different orders. The phylogenetic analyses of *Boletales* revealed that it can be divided into six major branches and the monophyly of *Boletaceae* was confirmed. However, three genera, namely, *Boletus*, *Retiboletus* and *Neoboletus*, were polyphyletic. The occurrence of *Neoboletus* species exhibiting distinct bruising discolouration patterns in phylogenetically separate clades suggests this morphological trait may correlate with underlying genetic divergence. In order to construct a clearer and more stable phylogenetic relationship of *Boletales*, it is necessary to include more species and accelerate the pace of sequencing in order to obtain diverse data.

## ﻿Additional information

### Use of AI

No use of AI was reported.

### Data availability

The data are available from the corresponding authors upon request.

## Supplementary materials

Supplementary material 1 Supplementary tables and figures Data type zip https://binary.pensoft.net/file/1395428This dataset is made available under the Open Database License (http://opendatacommons.org/licenses/odbl/1.0/). The Open Database License (ODbL) is a license agreement intended to allow users to freely share, modify, and use this Dataset while maintaining this same freedom for others, provided that the original source and author(s) are credited.Xianyi Wang, Jiawei Tao, Zhongyao Guo, Guoyu Wang, Guangyin Xu, Yaping Wang, Yaohang Long, Hongmei Liu
