# Supplementary material for: ﻿Comparative and phylogenetic analyses using mitogenomes revealed gene re-arrangement of Boletaceae (Boletales)
Source: IMA Fungus. 2025 Aug 15;16:e154192. doi: 10.3897/imafungus.16.154192 (PMC12374169; doi:10.3897/imafungus.16.154192)
Supplement: Supplementary material 1 — Supplementary tables and figures [file imafungus-16-e154192-s001.zip › supplementary file/supplementary file.docx]

| Species name | Genus | Locality | Altitude (m) | Longitude and latitude | Habitat and distribution | Time |
| --- | --- | --- | --- | --- | --- | --- |
| *A*. *granulopunctatus* | *Amoenoboletus* | Longli County, Guizhou Province | 1205.530 | 106.70°E, 26.37°N | Solitary on soils in coniferous forest | 2023.6.18 |
| *R. fuscus* | *Retiboletus* | Weining Yi and Hui and Miao Autonomous County, Guizhou Province | 1685.598 | 103.82°E, 27.09°N | Scattered on soils in mixed forest | 2023.7.28 |
| *L*. *parascabrum* | *Leccinum* | Longli County, Guizhou Province | Acquire | Acquire | Acquire | 2023.6.23 |
| *S*. *densisquamosus* | *Strobilomyces* | Longli County, Guizhou Province | 1209.383 | 106.60°E, 26.42°N | Solitary on soils in coniferous forest | 2023.6.18 |
| *B*. *bainiugan* | *Boletus* | Longli County, Guizhou Province | 1211.671 | 106.57°E, 26.34°N | Solitary on soils in coniferous forest | 2023.6.18 |
| *T*. *neofelleus* | *Tylopilus* | Longli County, Guizhou Province | 1207.562 | 106.93°E, 26.44°N | Scattered on soils in coniferous forest | 2023.6.3 |
| *H*. *nigropunctata* | *Hourangia* | Longli County, Guizhou Province | 1218.157 | 106.94°E, 26.43°N | Scattered on soils in coniferous forest | 2023.6.3 |

**Table S1:** Collection information of newly sequenced Boletaceae species.

**Table S2:** Information of samples for phylogenetic analysis.

| Family | Species | Genbank Number | Size (bp) | References |
| --- | --- | --- | --- | --- |
| Boletaceae | ***Amoenoboletus* *granulopunctatus*** | **PP048753** | 44,774 | **This study** |
|  | *Aureoboletus raphanaceus* | NC079662 | 42,157 | (Mu et al. 2024) |
|  | *Baorangia bicolor* | MW308599 | 38,082 | (Li et al. 2021) |
|  | ***Boletus bainiugan*** | **PP048754** | 34,893 | **This study** |
|  | *Boletus edulis* | MW308609 | 34,763 | (Li et al. 2021) |
|  | *Boletus* sp1 | MW308606 | 35,304 | (Li et al. 2021) |
|  | *Boletus* sp2 | MW308608 | 48,298 | (Li et al. 2021) |
|  | *Boletus speciosus* | MW308600 | 36,622 | (Li et al. 2021) |
|  | *Butyriboletus roseoflavus* | MZ202357 | 36,551 | Unpublished |
|  | *Caloboletus calopus* | MW308602 | 32,883 | (Li et al. 2021) |
|  | *Hemileccinum impolitum* | NC056808 | 39,362 | Unpublished |
|  | ***Hourangia nigropunctata*** | **PP048751** | 32,910 | **This study** |
|  | *Lanmaoa macrocarpa* | NC080885 | 38,139 | (Zheng et al. 2024) |
|  | ***Leccinum parascabrum*** | **PP048752** | 41,354 | **This study** |
|  | *Neoboletus brunneissimus* | MW308605 | 42,147 | (Li et al. 2021) |
|  | *Neoboletus magnificus* | MW308603 | 39,449 | (Li et al. 2021) |
|  | *Neoboletus obscureumbrinus* | MW308607 | 39,929 | (Li et al. 2021) |
|  | *Neoboletus subvelutipes* | MW308604 | 34,668 | (Li et al. 2021) |
|  | *Pulveroboletus ravenelii* | NC061666 | 43,528 | (Cho et al. 2022) |
|  | ***Retiboletus fuscus*** | **PP048756** | 40,302 | **This study** |
|  | *Retiboletus ornatipes* | MW308601 | 36,785 | (Li et al. 2021) |
|  | *Rufoboletus hainanensis* | NC082970 | 36,592 | (Zeng et al. 2024) |
|  | ***Strobilomyces densisquamosus*** | **PP048755** | 36,132 | **This study** |
|  | *Tylopilus brunneirubens* | NC084291 | 32,389 | (Huang et al. 2024) |
|  | ***Tylopilus neofelleus*** | **PP048750** | 33,453 | **This study** |
|  | *Tylopilus plumbeoviolaceoides* | NC056835 | 37,242 | (Shi et al. 2022) |
| Paxillaceae | *Paxillus involutus* | NC045203 | 39,109 | (Li et al. 2020) |
|  | *Paxillus rubicundulus* | NC045204 | 41,061 | (Li et al. 2020) |
| Boletinellaceae | *Phlebopus portentosus* | MK571437 | 43,299 | (Jiang et al. 2017) |
| Sclerodermataceae | *Pisolithus microcarpus* | NC054201 | 43,990 | (Wu et al. 2017) |
|  | *Pisolithus tinctorius* | NC054202 | 44,054 | (Wu et al. 2017) |
| Rhizopogonaceae | *Rhizopogon salebrosus* | NC042698 | 66,704 | (Li et al. 2019a) |
|  | *Rhizopogon vinicolor* | NC042699 | 77,109 | (Li et al. 2019a) |
| Gomphidiaceae | *Chroogomphus rutilus* | MZ151416 | 37,508 | (Fu et al. 2021) |
| Outgroups | *Ganoderma lingzhi* | NC062658 | 56,044 | (Li et al. 2019b) |
|  | *Trametes coccinea* | NC054272 | 99,976 | (Chen et al. 2021) |

**
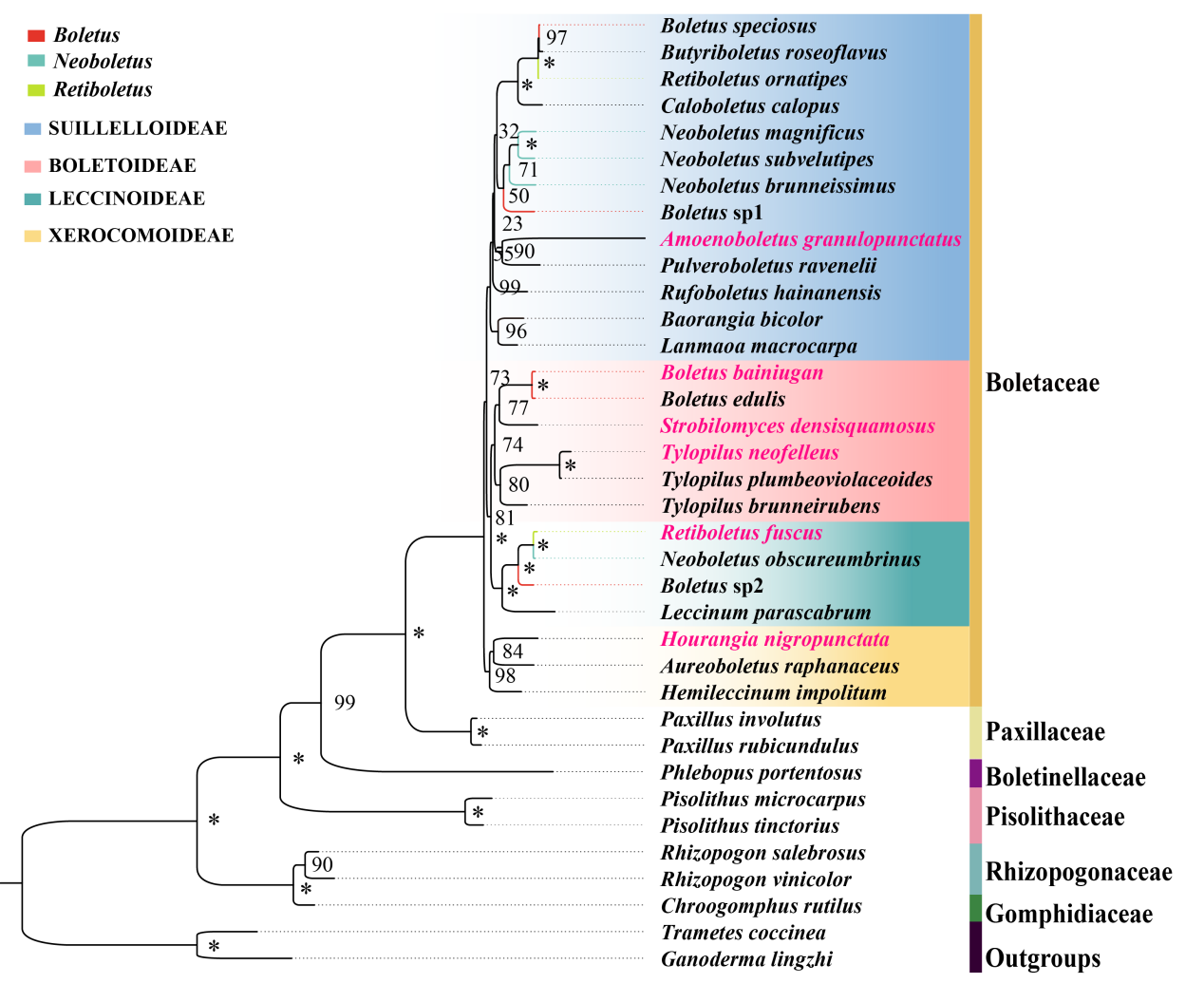
**

**Fig. S1** Phylogenetic relationships of 34 Boletales based on AA using IQ-TREE. The asterisk indicates BPP = 1. The colored lines indicate that the genus is polyphyletic. The seven newly sequenced mitogenomes are highlighted in pink.

**
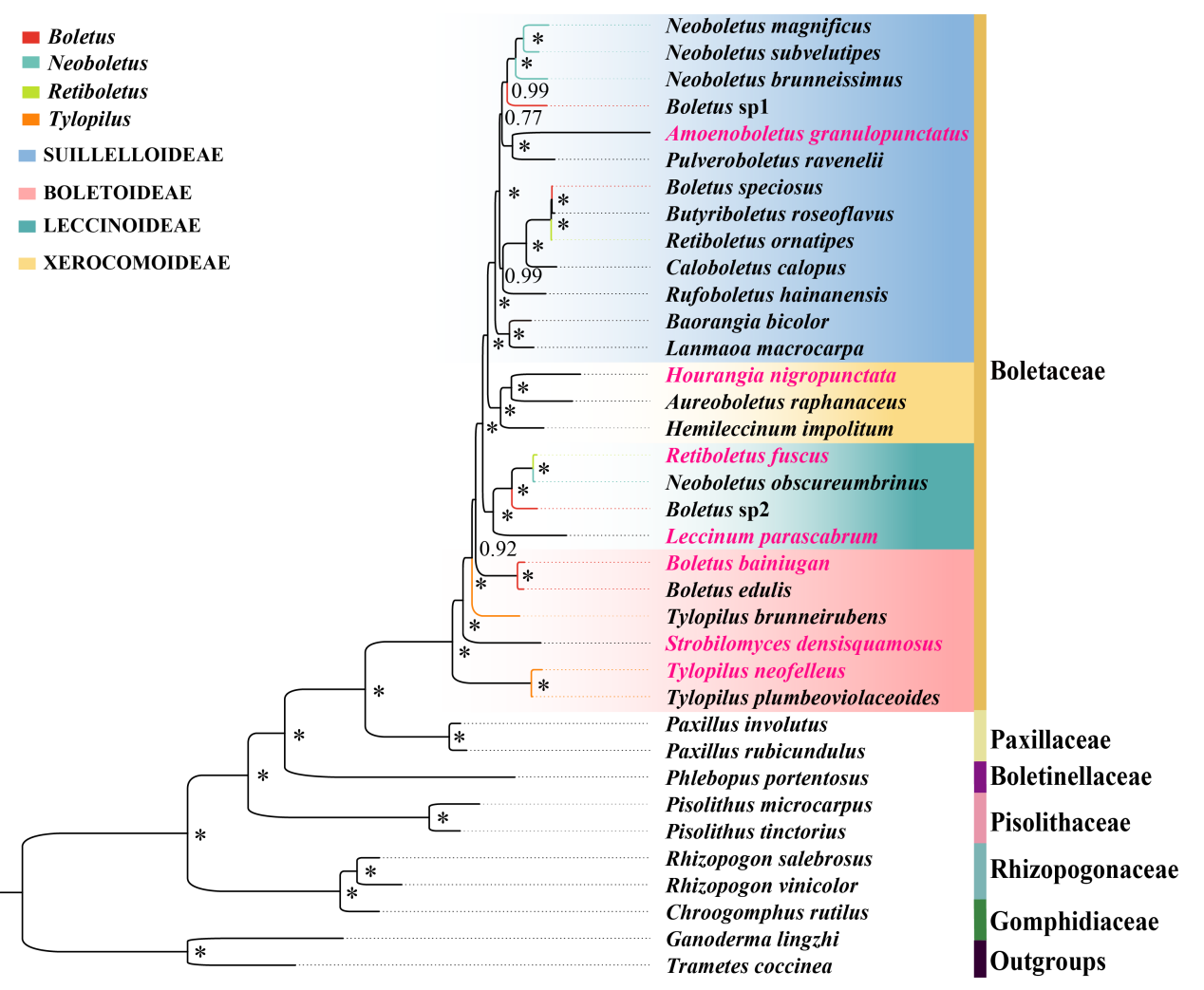
**

**Fig. S2** Phylogenetic relationships of 34 Boletales based on PCG using MrBayes. The asterisk indicates BPP = 1. The colored lines indicate that the genus is polyphyletic. The seven newly sequenced mitogenomes are highlighted in pink.


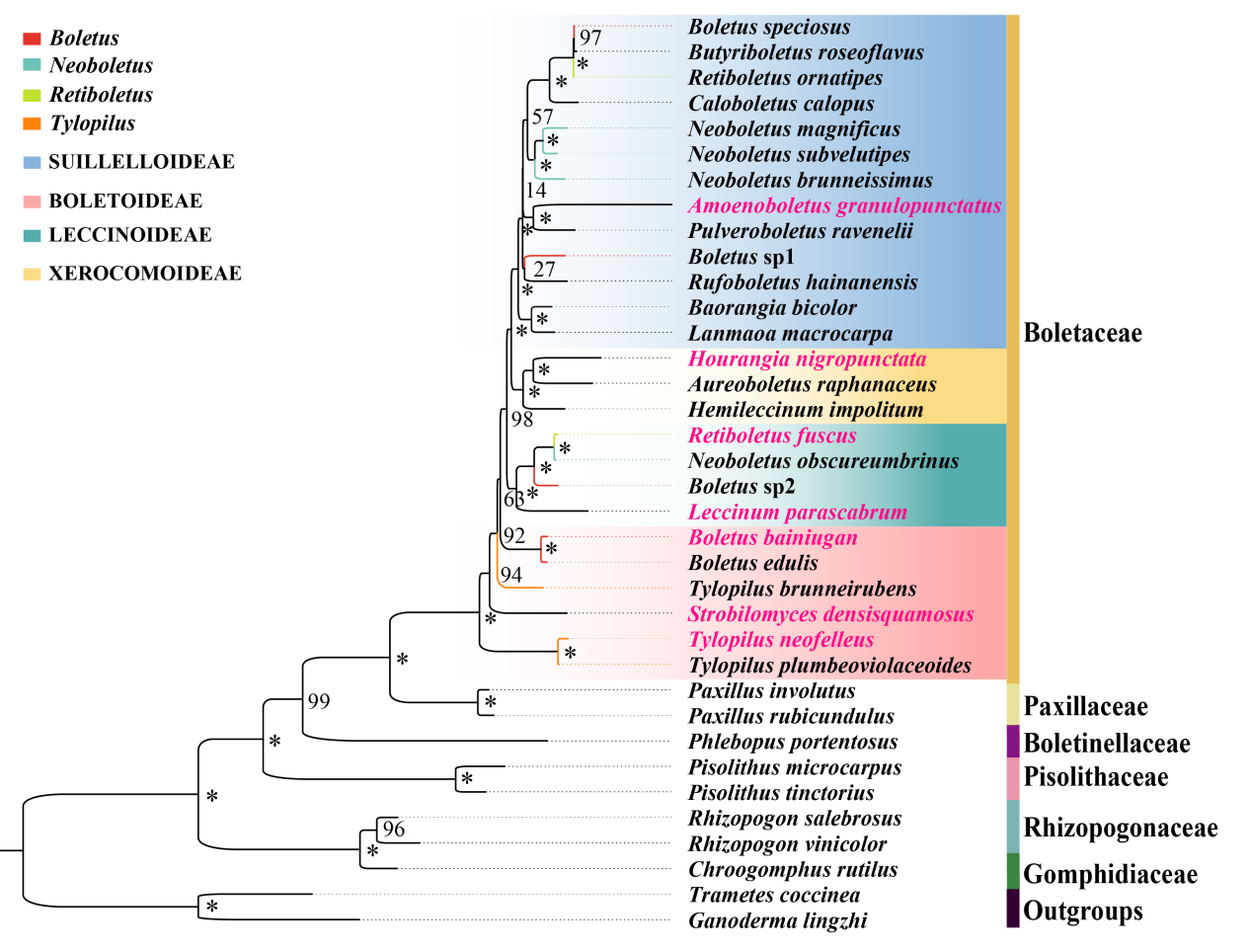


**Fig. S3** Phylogenetic relationships of 34 Boletales based on PCG using IQ-TREE. The asterisk indicates BS = 100. The colored lines indicate that the genus is polyphyletic. The seven newly sequenced mitogenomes are highlighted in pink.


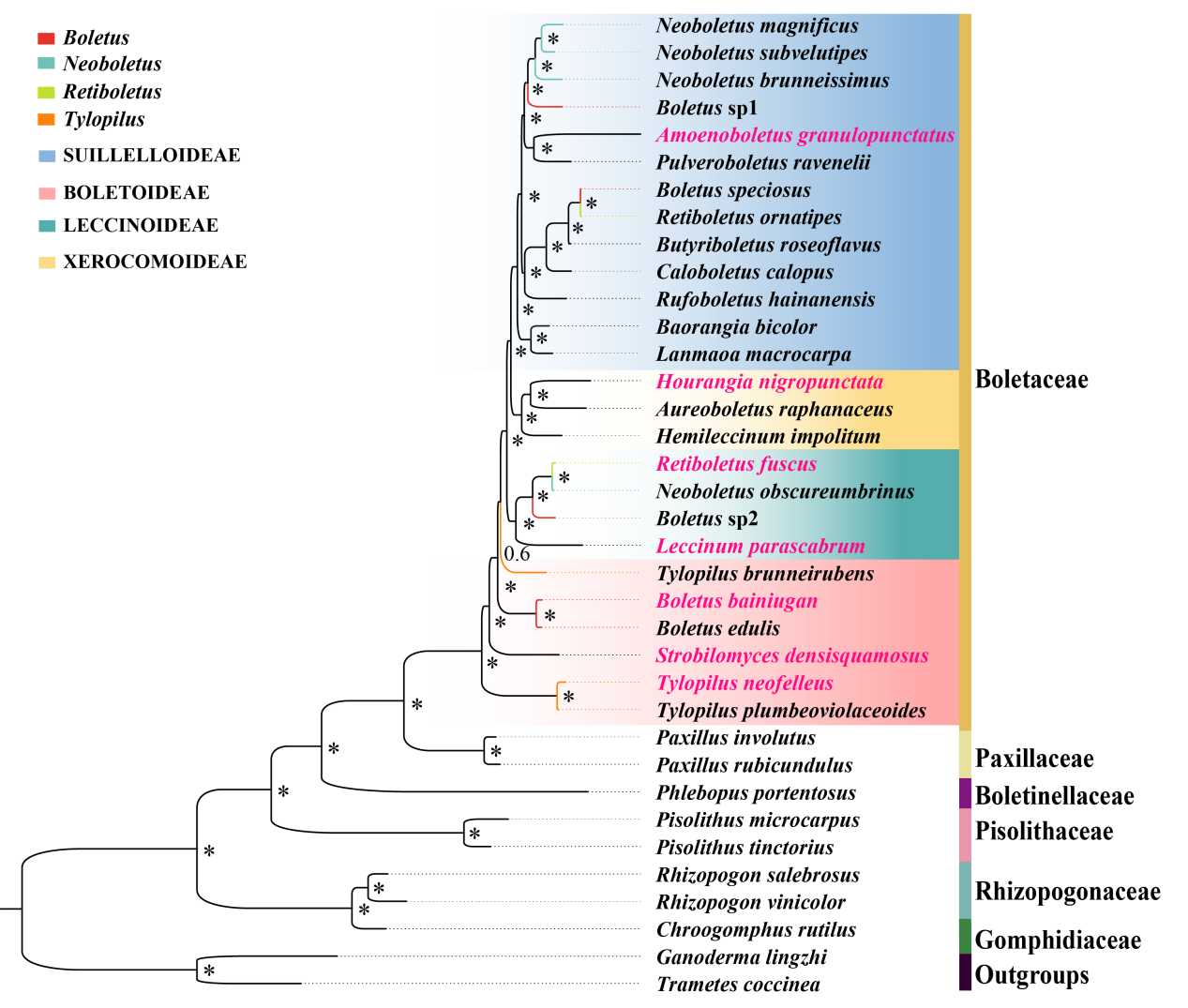


**Fig. S4** Phylogenetic relationships of 34 Boletales based on PCGRNA using MrBayes. The asterisk indicates BPP = 1. The colored lines indicate that the genus is polyphyletic. The seven newly sequenced mitogenomes are highlighted in pink.


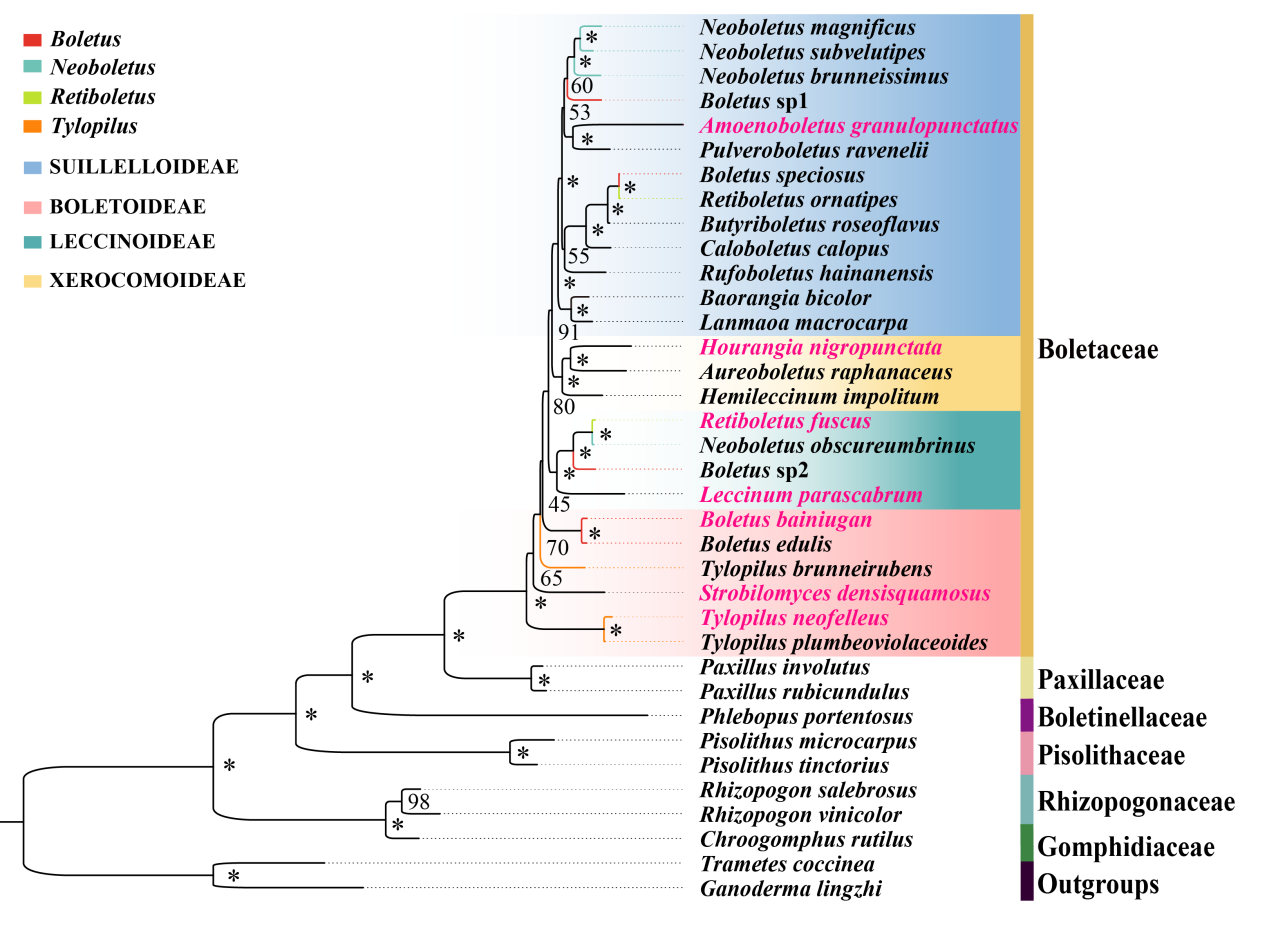


**Fig. S5** Phylogenetic relationships of 34 Boletales based on PCGRNA using IQ-TREE. The asterisk indicates BS = 100. The colored lines indicate that the genus is polyphyletic. The seven newly sequenced mitogenomes are highlighted in pink.


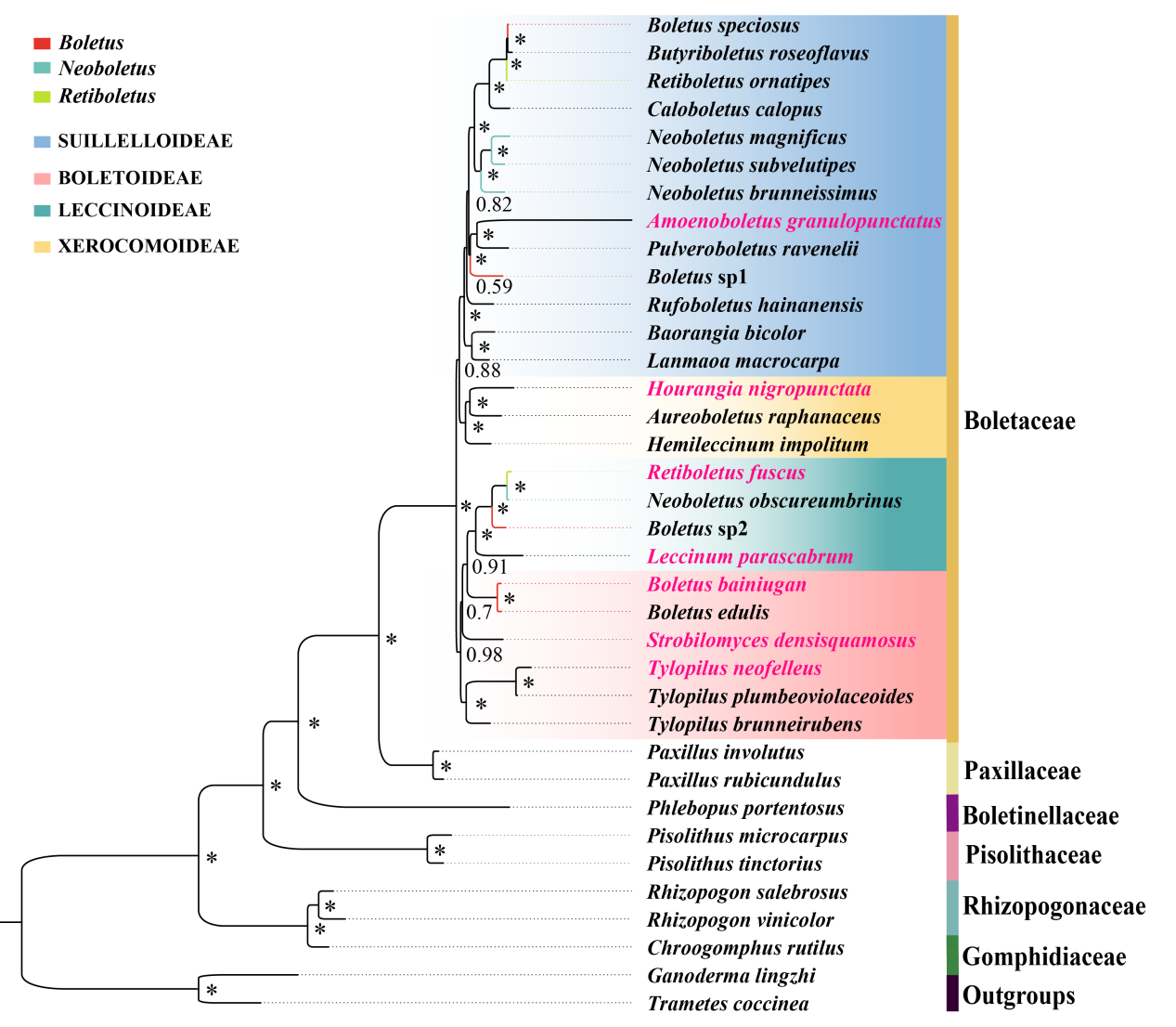


**Fig. S6** Phylogenetic relationships of 34 Boletales based on PCG12 using MrBayes. The asterisk indicates BPP = 1. The colored lines indicate that the genus is polyphyletic. The seven newly sequenced mitogenomes are highlighted in pink.


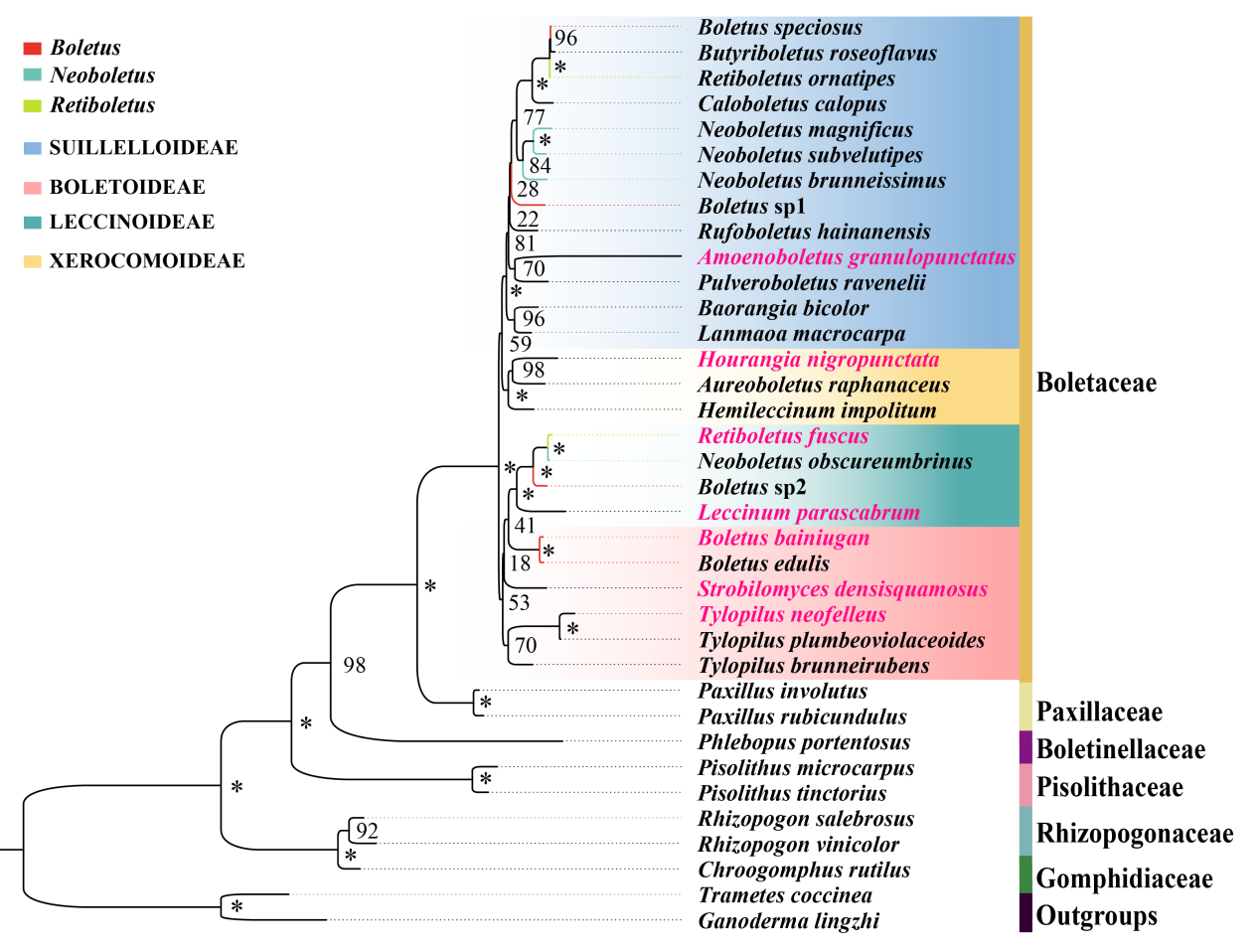


**Fig. S7** Phylogenetic relationships of 34 Boletales based on PCG12 using IQ-TREE. The asterisk indicates BS = 100. The colored lines indicate that the genus is polyphyletic. The seven newly sequenced mitogenomes are highlighted in pink.


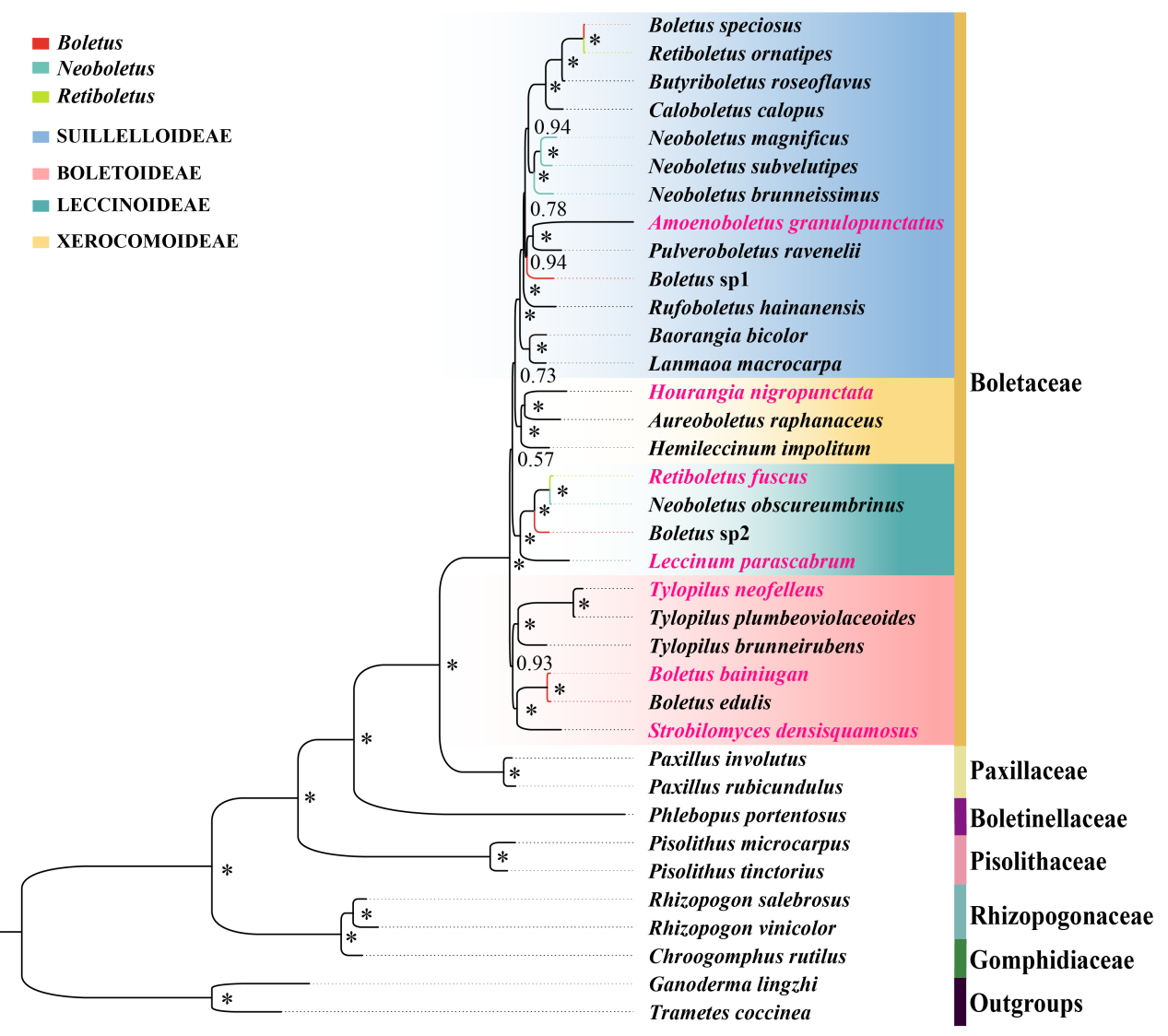


**Fig. S8** Phylogenetic relationships of 34 Boletales based on PCG12RNA using MrBayes. The asterisk indicates BPP = 1. The colored lines indicate that the genus is polyphyletic. The seven newly sequenced mitogenomes are highlighted in pink.


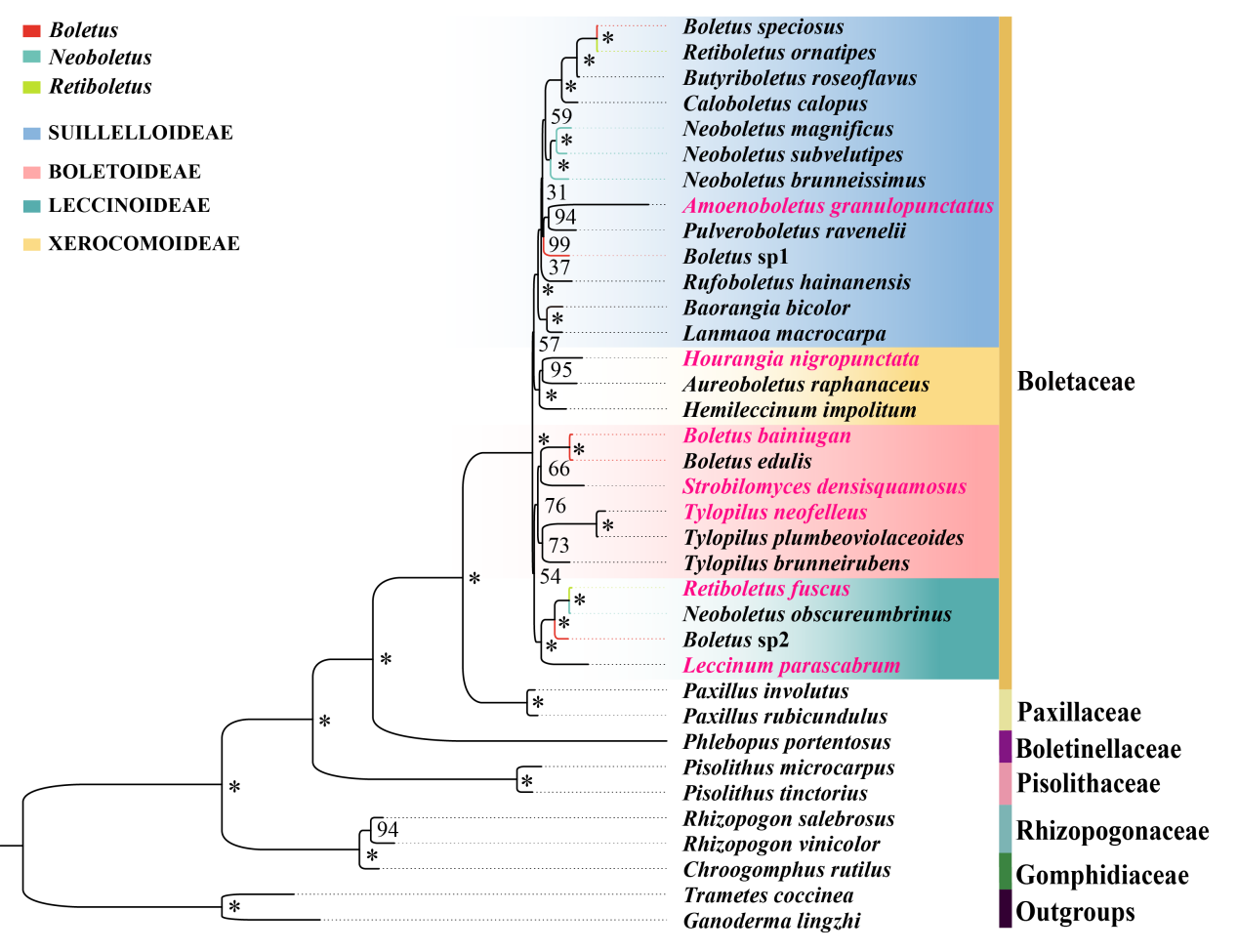


**Fig. S9** Phylogenetic relationships of 34 Boletales based on PCG12RNA using IQ-TREE. The asterisk indicates BS = 100. The colored lines indicate that the genus is polyphyletic. The seven newly sequenced mitogenomes are highlighted in pink.
