# Supplementary figures and images for: ﻿Comparative and phylogenetic analyses using mitogenomes revealed gene re-arrangement of Boletaceae (Boletales)
Source: IMA Fungus. 2025 Aug 15;16:e154192. doi: 10.3897/imafungus.16.154192 (PMC12374169; doi:10.3897/imafungus.16.154192)

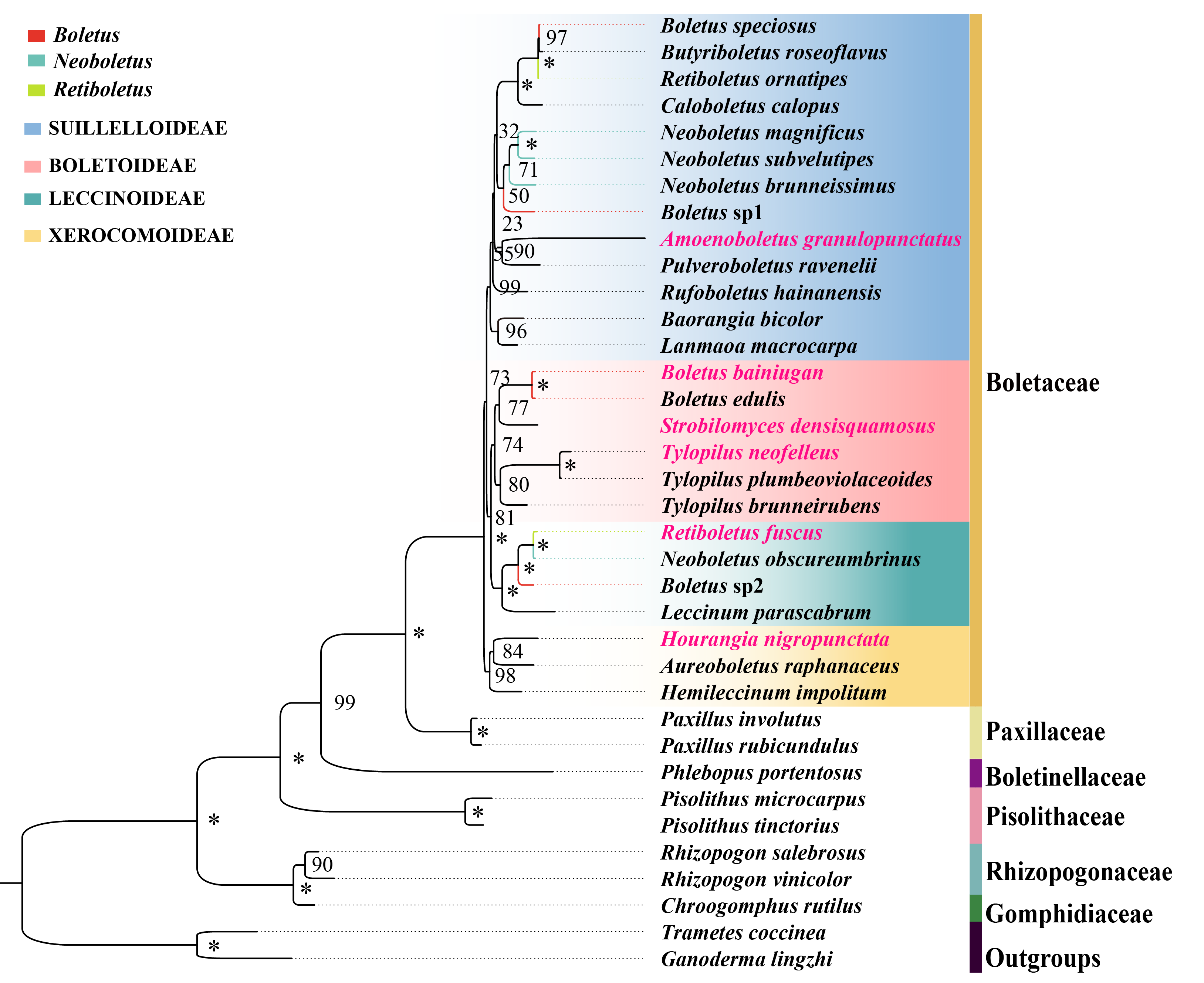

Supplement: Supplementary material 1 — Supplementary tables and figures [file imafungus-16-e154192-s001.zip › supplementary file/Fig. S1.png]

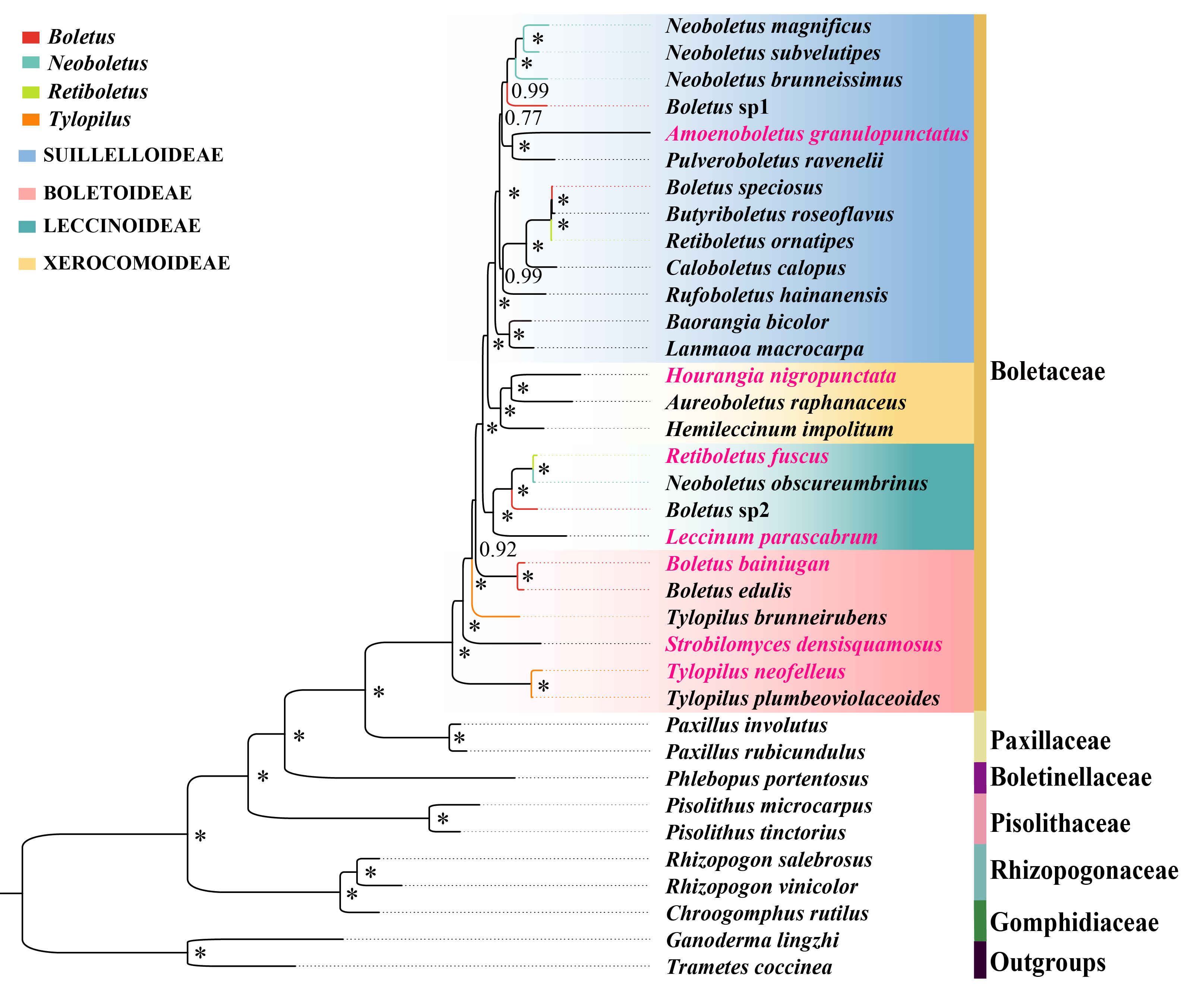

Supplement: Supplementary material 1 — Supplementary tables and figures [file imafungus-16-e154192-s001.zip › supplementary file/Fig. S2.png]

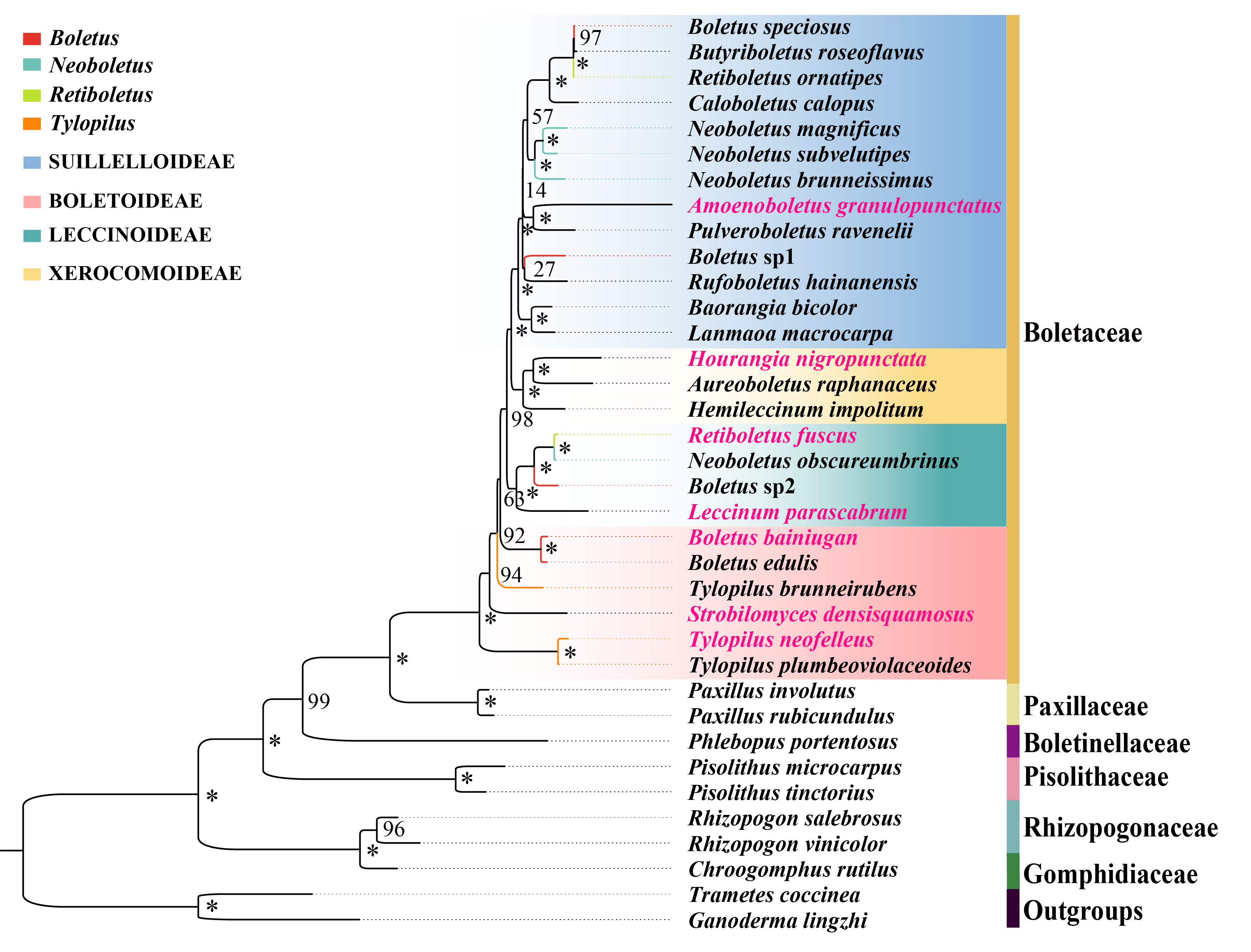

Supplement: Supplementary material 1 — Supplementary tables and figures [file imafungus-16-e154192-s001.zip › supplementary file/Fig. S3.png]

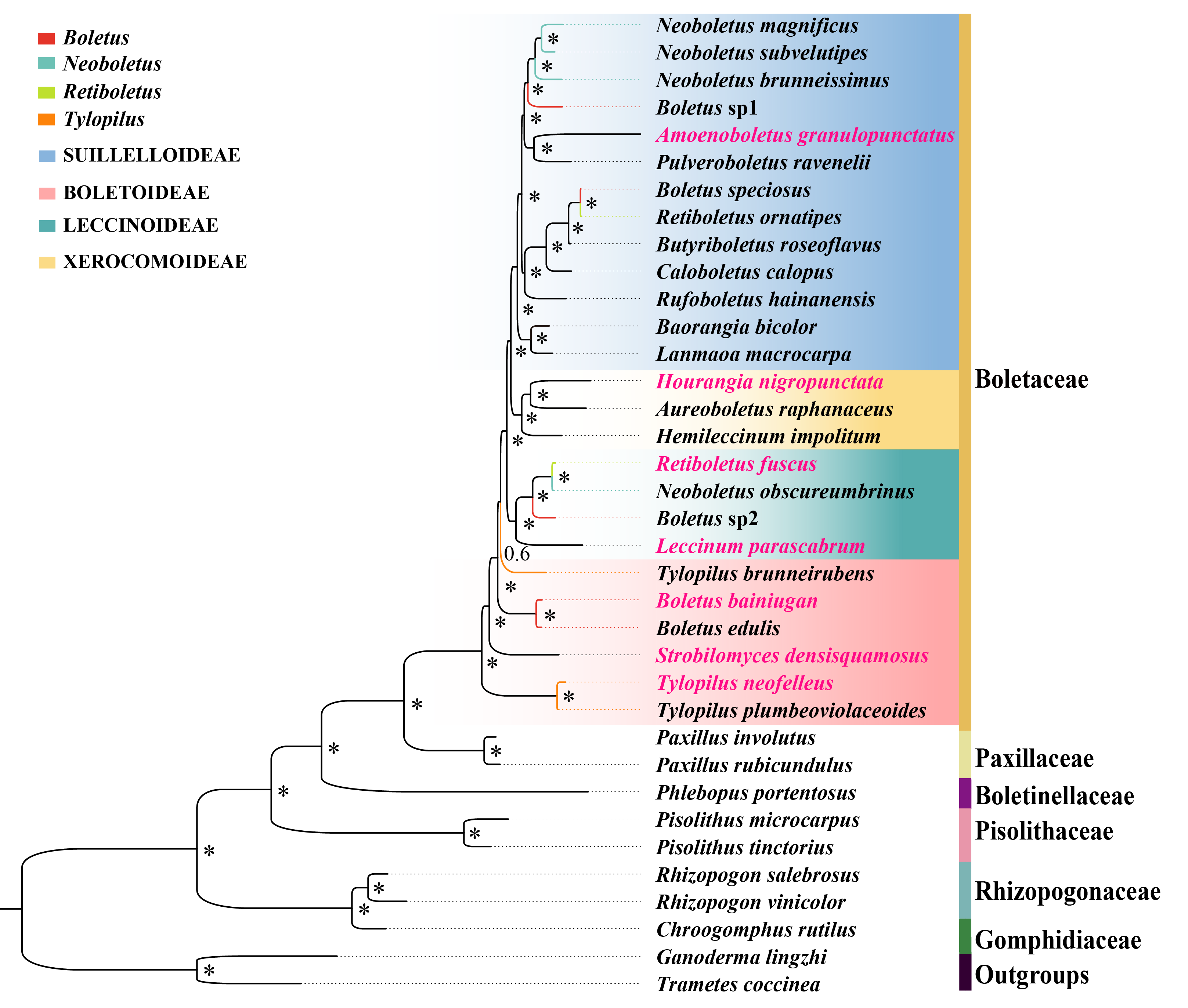

Supplement: Supplementary material 1 — Supplementary tables and figures [file imafungus-16-e154192-s001.zip › supplementary file/Fig. S4.png]

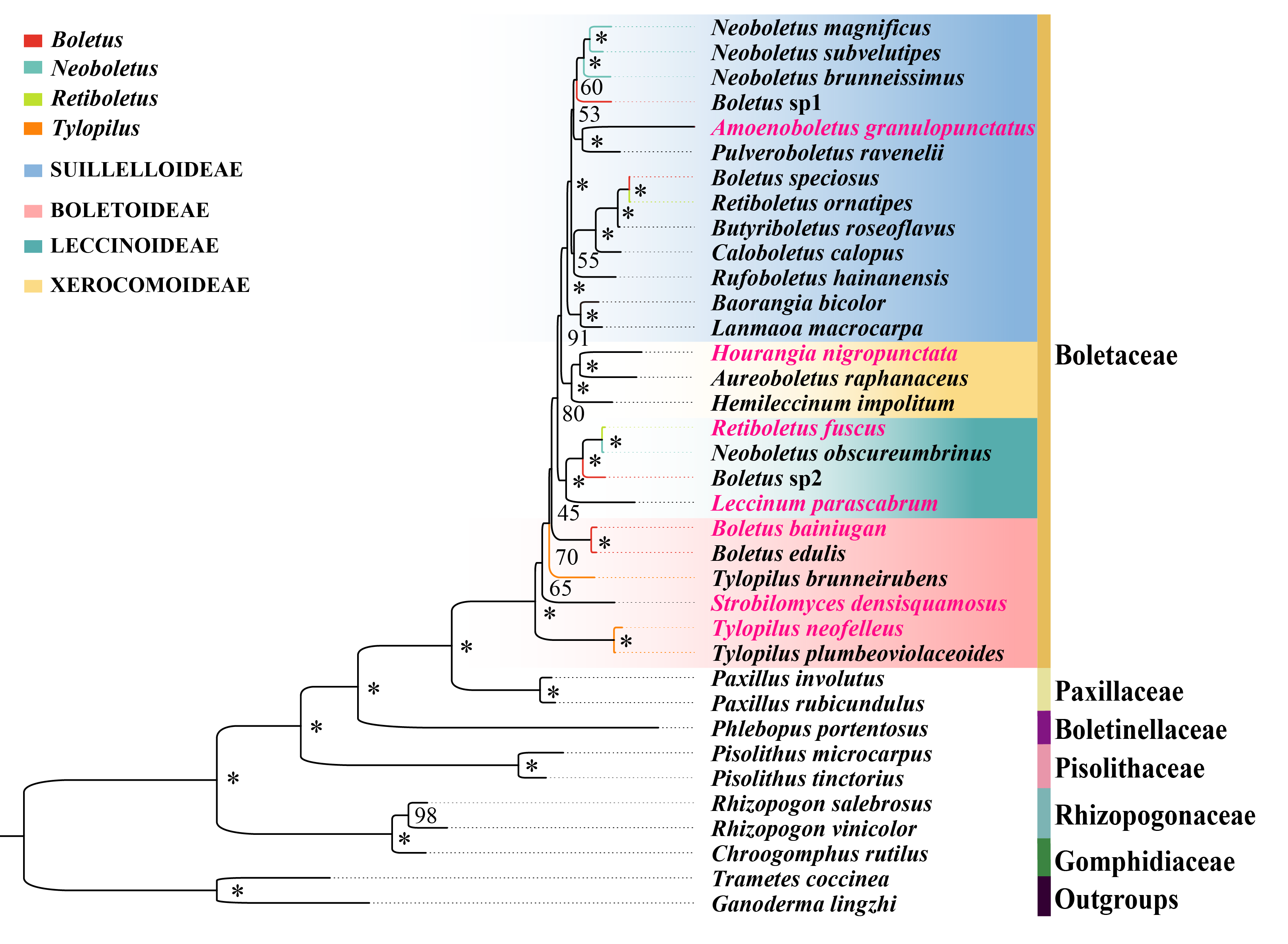

Supplement: Supplementary material 1 — Supplementary tables and figures [file imafungus-16-e154192-s001.zip › supplementary file/Fig. S5.png]

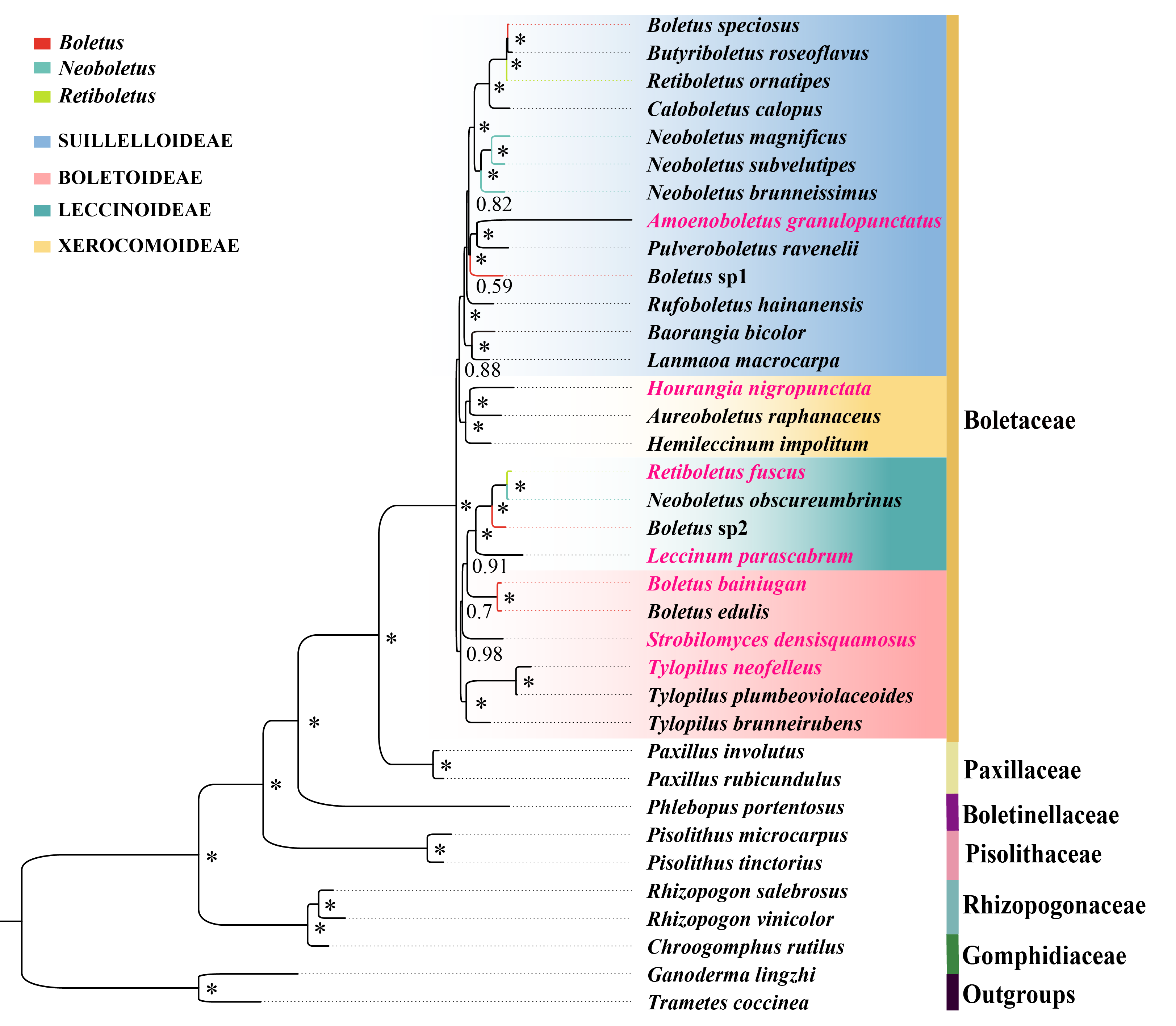

Supplement: Supplementary material 1 — Supplementary tables and figures [file imafungus-16-e154192-s001.zip › supplementary file/Fig. S6.png]

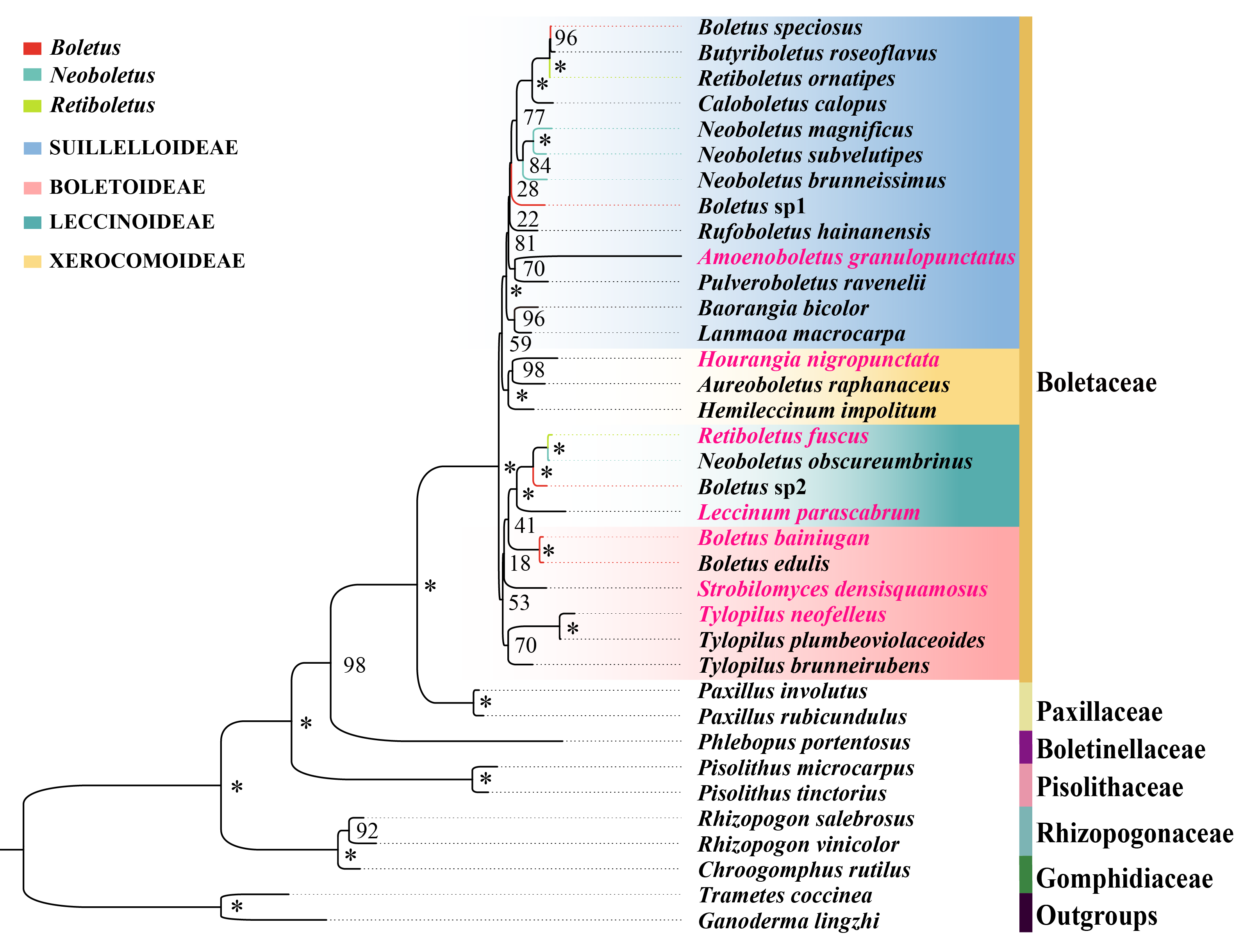

Supplement: Supplementary material 1 — Supplementary tables and figures [file imafungus-16-e154192-s001.zip › supplementary file/Fig. S7.png]

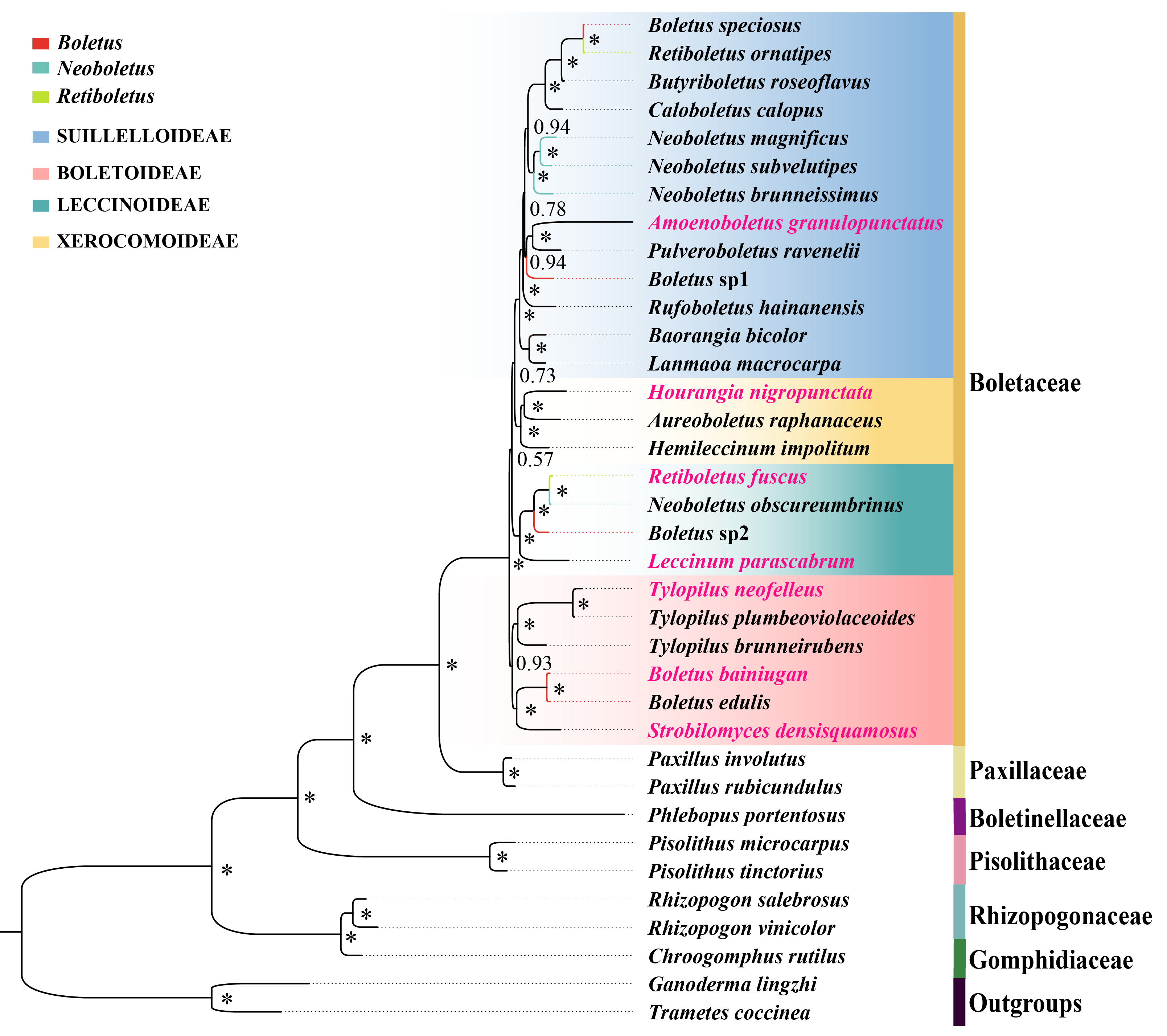

Supplement: Supplementary material 1 — Supplementary tables and figures [file imafungus-16-e154192-s001.zip › supplementary file/Fig. S8.png]

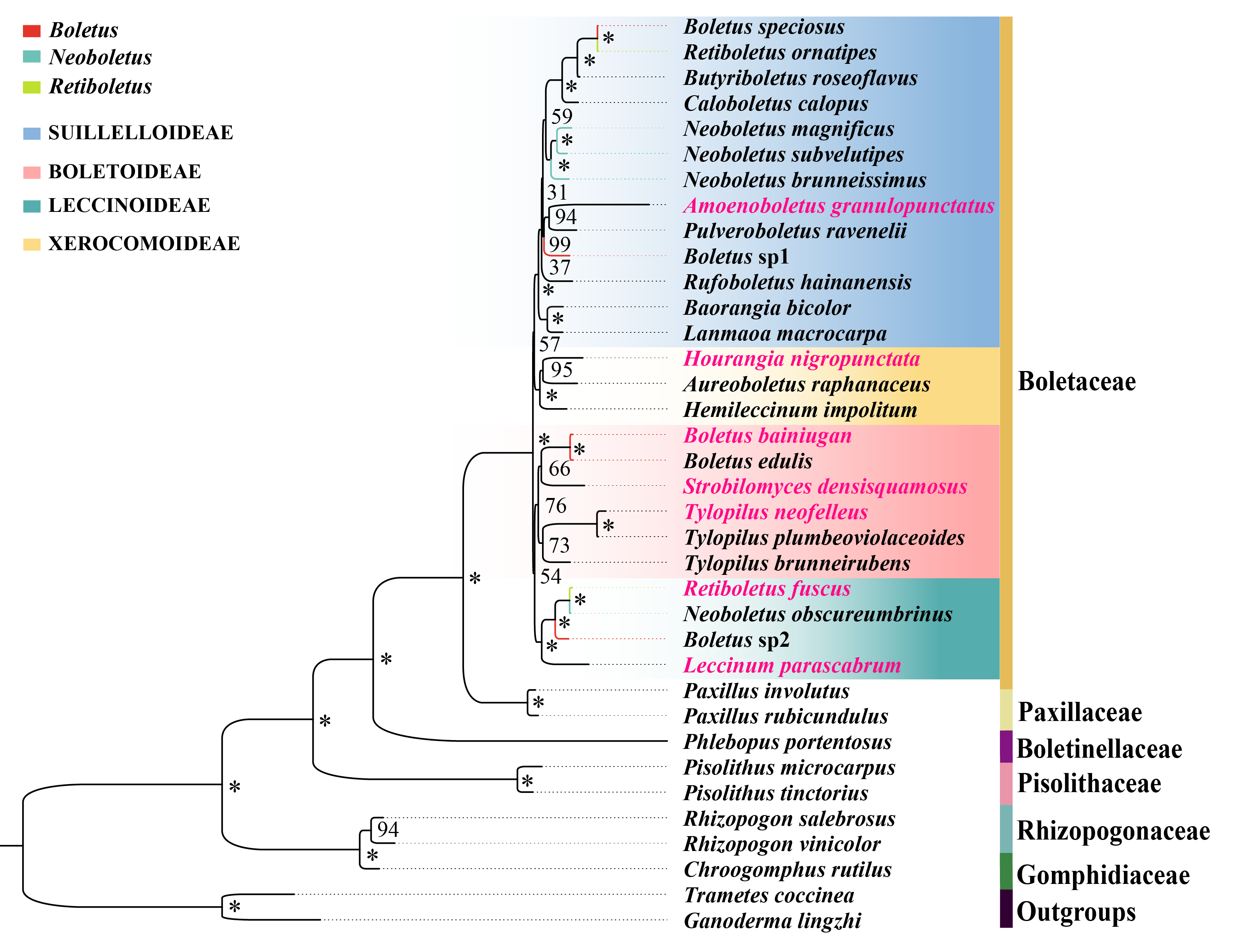

Supplement: Supplementary material 1 — Supplementary tables and figures [file imafungus-16-e154192-s001.zip › supplementary file/Fig. S9.png]
